# An overview on neurobiology and therapeutics of attention-deficit/hyperactivity disorder

**DOI:** 10.1007/s44192-022-00030-1

**Published:** 2023-01-05

**Authors:** Bruna Santos da Silva, Eugenio Horacio Grevet, Luiza Carolina Fagundes Silva, João Kleber Neves Ramos, Diego Luiz Rovaris, Claiton Henrique Dotto Bau

**Affiliations:** 1 ADHD and Developmental Psychiatry Programs, Hospital de Clínicas de Porto Alegre, Universidade Federal Do Rio Grande Do Sul, Porto Alegre, Brazil; 2 Department of Genetics and Graduate Program in Genetics and Molecular Biology, Instituto de Biociências, Universidade Federal Do Rio Grande Do Sul, Porto Alegre, Brazil; 3 Department of Psychiatry and Graduate Program in Psychiatry and Behavioral Sciences, Faculdade de Medicina, Universidade Federal Do Rio Grande Do Sul, Porto Alegre, Brazil; 4 Department of Physiology and Biophysics, Instituto de Ciencias Biomedicas Universidade de Sao Paulo, São Paulo, Brazil; 5 Laboratory of Physiological Genomics of Mental Health (PhysioGen Lab), Institute of Biomedical Sciences, University of Sao Paulo, São Paulo, Brazil

## Abstract

Attention-Deficit/Hyperactivity Disorder (ADHD) is a prevalent psychiatric condition characterized by developmentally inappropriate symptoms of inattention and/or hyperactivity/impulsivity, which leads to impairments in the social, academic, and professional contexts. ADHD diagnosis relies solely on clinical assessment based on symptom evaluation and is sometimes challenging due to the substantial heterogeneity of the disorder in terms of clinical and pathophysiological aspects. Despite the difficulties imposed by the high complexity of ADHD etiology, the growing body of research and technological advances provide good perspectives for understanding the neurobiology of the disorder. Such knowledge is essential to refining diagnosis and identifying new therapeutic options to optimize treatment outcomes and associated impairments, leading to improvements in all domains of patient care. This review is intended to be an updated outline that addresses the etiological and neurobiological aspects of ADHD and its treatment, considering the impact of the “omics” era on disentangling the multifactorial architecture of ADHD.

**Supplementary Information:**

The online version contains supplementary material available at 10.1007/s44192-022-00030-1.

## Introduction

Attention-Deficit/Hyperactivity Disorder (ADHD) is characterized by developmentally inappropriate symptoms of inattention and/or hyperactivity/impulsivity, which leads to impairments in the social, school/academic, and professional contexts. The worldwide prevalence of ADHD is among the highest for neurodevelopmental disorders, estimated at ≈5–7% for children/adolescents [1, 2] and ≈2.5% for adults [3, 4].

ADHD diagnosis requires a careful clinical assessment based on symptom evaluation and functional impairments. Diagnostic classification practices such as the Diagnostic and Statistical Manual of Mental Disorders—5th edition (DSM-5; APA 2013) offer critical standard criteria to guide clinicians for an accurate diagnosis. According to DSM-5, to fulfill the diagnosis criteria, patients should present: (a) at least six (for children and adolescents) or five (for adults) out of the nine symptoms of inattention and/or hyperactivity/impulsivity for a minimum of 6 months; (b) symptoms onset before 12 years of age; (c) symptoms that cause impairments in living; (d) symptoms that affect different life contexts (e.g., home and school). Notably, the manifestation of symptoms should not be better explained by other conditions (e.g., schizophrenia, bipolar disorder, etc.). DSM-5 describes three clinical presentations of ADHD: predominantly inattentive, predominantly hyperactive, or combined. In general, the inattentive and combined presentations are the most frequently observed among patients, except for young children (3–5 years old), for which hyperactivity is more pronounced [5, 6].

ADHD diagnosis is sometimes challenging due to the substantial heterogeneity of the disorder in terms of clinical and pathophysiological aspects, a fact that impacts research on the etiological and neurobiological specificities of ADHD [7]. The high rates of psychiatric comorbidities observed in patients with ADHD and the considerable proportion of overlap in symptoms and causes with other mental disorders are key factors to be considered in this sense. More than 60% of individuals with ADHD present at least one comorbid psychiatric disorder, frequently including depressive, anxiety, and disruptive behavioral disorders [8–10]. Autism spectrum disorder (ASD) and ADHD are also frequently comorbid [11, 12]. According to the current DSM-5 diagnostic criteria, ADHD diagnosis should no longer be excluded in the presence of ASD, as in previous versions. Patients with ADHD also present higher rates of obesity, sleep disturbances, asthma, autoimmune and inflammatory diseases, and other somatic and metabolic-related problems [13–15].

Another source of heterogeneity is the diverse clinical profile of children and adults with ADHD. For example, the comorbidity profile mentioned above also varies across development. In children, the most common comorbidities include externalizing (conduct and oppositional defiant) and learning disorders. In contrast, mood, anxiety, and especially personality and substance use disorders become more prevalent in adult life [13, 14]. In addition, while in children and adolescents ADHD is more frequent in boys than in girls, ranging from 3:1 to 10:1 ratios, in adulthood, a more balanced distribution of prevalence across genders is observed [16, 17]. Beyond the age-dependent decline of symptoms, especially in the hyperactive/impulsive domain [18], clinical follow-ups have shown that ADHD can fluctuate or remit throughout the lifespan, including during adulthood [19–22]. Population cohort studies have also demonstrated that the adult ADHD population may not be composed only of individuals with ADHD initiating in childhood and persisting into adulthood but also comprised by at least 20% of late-onset cases occurring in late adolescence and young adulthood [23–26], challenging the classical neurodevelopmental conceptualization of the disorder [27].

Considering the consequences of ADHD symptomatology in different life contexts and its high social and economic impact, the clinical, epidemiological, and neurobiological aspects of this disorder have been widely studied. Despite the challenges related to clinical heterogeneity, as mentioned above, promising perspectives to understanding the neurobiological basis of ADHD have emerged in the last few years. In this literature review, we provide an updated appraisal of the etiological and neurobiological aspects of ADHD and its treatment in light of the “omics” era for disentangling the multifactorial architecture of ADHD. The search was based on published research collected from the PubMed database using general search keywords for each topic followed by a critical evaluation of existing research to include the most relevant evidence (i.e., supported by meta-analysis, highlighted by systematic reviews and/or large recent studies presenting robust and unbiased methodologies).

## Environmental correlates of ADHD

Among the environmental variables proposed as risk factors predisposing to ADHD, pre-, peri- and postnatal factors are the most frequently associated, including low birth weight and prematurity [28], maternal exposure to stressful events, or other health-related maternal conditions [29, 30]. Significant effects of maternal (during pregnancy) and/or early childhood exposures to cigarettes [31], alcohol [32], medications [33–35], and other substances such as lead and manganese [36] on the risk of ADHD have been reported. See Additional file 1: Tables S1 and S2 for a comprehensive overview of meta-analyses reporting the effects of prenatal, newborn, and lifespan-related factors and exposures. Nutritional aspects and diet across the lifespan have also been considered as factors that potentially influence ADHD susceptibility [37–39]. See Additional file 1: Table S3 for more detailed information from meta-analyses results investigating biomarkers and nutritional factors. Other risk factors for ADHD include psychosocial conditions such as low income, hostile parental care, and life adversities. However, it is important to consider that these factors might have more prominent effects as course modifiers of prognosis rather than in the predisposition of the disorder [40].

Although investigations on environmental risk factors for ADHD have yielded some reproducible findings, causality inferences remain elusive since unmeasured factors may confound the results [41]. The potential effects of gene-environment interactions and correlations should be considered. In this regard, a possible effect of the genetic variation on the predisposition of exposure to specific risk or protective environmental contexts may indirectly affect ADHD susceptibility, imposing difficulties in interpreting associations [42]. Maternal-related risk factors, for example, may be confounded by familial or genetic factors. On the other hand, some environmental exposures can also result in biological modifications, such as DNA methylation, which are reversible changes in genomic function independent of the DNA sequence that has also been associated with ADHD [43, 44]. Thus, environment and genetics act together in the etiology of ADHD, making it challenging to disentangle several sources of bias/confounding, possibly affecting the observed associations of environmental risk factors.

## Genetics of ADHD

ADHD has a multifactorial etiology, in which genetic and environmental factors are involved in its development. The genetic contribution to ADHD is among the highest for psychiatric disorders. A high family aggregation is reported for ADHD and data from a large population sample demonstrated that the hazard ratio for ADHD relatives, compared to the frequency of ADHD in relatives of unaffected people, is 70 for monozygotic twins, around 8 for dizygotic twins and full siblings, between 2 and 3 for half-siblings, 2.2 for full cousins, and 1.5 for half cousins [45]. The total heritability of ADHD has been estimated at 70–80% [46, 47]. These estimates seem stable throughout childhood and adolescence [48] and similar for both males and females [49]. For adults, some studies, especially those based on self-report measures, describe a drop in heritability estimates; however, studies that used cross-informant data and/or clinical diagnosis report that heritability estimates in adults could be as high as those observed in children, suggesting that phenotype measurement biases could play a larger role than true age-dependent decline [50]. Although additive effects are the major component of heritability, available evidence suggests that nonadditive effects (genetic interactions) also contribute to a large proportion of phenotypic variance and that the effects of a shared family environment are minimal [49, 51].

The understanding of the genetic architecture of ADHD has significantly evolved with the technological advances in the molecular genetics field, especially with the availability of large-scale genome-wide association studies (GWAS). Based on these studies, the proportion of the phenotypic variation attributed to common genetic variants (known as h^2^_SNP_) has been estimated for ADHD. The ADHD GWAS that reported the first genome-wide significant findings (2019-ADHD) estimated the ADHD h^2^_SNP_ at ≈22% [52]. In an updated GWAS meta-analysis of ADHD (2022-ADHD), the h^2^_SNP_ was lower than in the previous GWAS, valued at 14% [53]. The authors attributed this difference to varying ascertainment strategies and designs or lower effective sample sizes on specific individual cohorts that comprised the study. Nevertheless, the results indicate that around 7000 common variants can explain 90% of the h^2^_SNP_, confirming the extensive polygenic architecture of ADHD and that many hits, in addition to those already discovered, will become significant as the sample size increases. Although the h^2^_SNP_ estimated for ADHD is far from reaching the heritability estimated in twin studies, it should be noticed that the h^2^_SNP_ of psychiatric phenotypes is usually much lower than the total heritability because of unmeasured factors in GWAS, such as rare and structural variants, gene–gene and gene-environmental interactions, and epigenomics.

The 2019-ADHD GWAS comprised a sample of 20,183 cases and 35,191 controls (the first wave of data from the Danish iPSYCH15 cohort (iPSYCH1) plus 11 ADHD cohorts collected by the Psychiatric Genomics Consortium (PGC)) and revealed 12 significant hits. The 2022-ADHD GWAS, including the extended Danish iPSYCH cohort (iPSYCH1 plus new iPSYCH2 data), the Icelandic deCODE cohort, and the PGC, totalizing a sample of 38,691 individuals with ADHD and 186,843 controls, showed 27 genome-wide loci associated with ADHD, of which 21 were new [53]. The associated loci include variants located around genes involved in a variety of processes, such as neurodevelopmental mechanisms and neurogenesis (e.g., *PPP1R16A*, *B4GALT2, PTPRF*, *SORCS3*, *DUSP6*, *FOXP2*, *MEF2C*, and *METTL15* genes).

Initially, it was expected the first significant hits to be revealed in these GWAS would be dopaminergic and noradrenergic genes, which were previously implicated in ADHD by meta-analysis of candidate gene studies, such as those encoding the dopamine receptors (e.g. *DRD2*, *DRD4*), the dopamine transporter (*DAT1*) and the enzymes monoamine oxidase (*MAOA*), catechol-O-methyltransferase (*COMT*) and dopamine beta-hydroxylase (*DBH*) [54, 55]. Interestingly, these GWAS pointed out none of such candidate genes previously implicated in ADHD. However, as mentioned above, the h^2^_SNP_ is explained by a much higher number of SNPs than those significantly associated. Therefore, genetic variants pointed out by candidate genes can still become significant as the sample size increases, as observed for other disorders, such as schizophrenia and depression, for which the significance in variants of candidate genes only emerged when GWAS reached huge sample sizes (> 150 thousand individuals) [56–58].

Genomic studies revealed an extensive genetic overlap of ADHD with a wide range of phenotypes, including psychiatric, cognitive, behavioral, and metabolic domains, suggesting that the clinically observed association of ADHD with these phenotypes can be explained, at least in part, by shared genetic factors [52, 53, 59]. The cross-trait genetic correlation is usually estimated using the linkage disequilibrium score regression (LDSC) or the polygenic scores (PGS) approaches. These analyses have revealed negative relationships between ADHD and different neurocognitive domains, including attention (beta = − 0.07, P = 3,25E-7) and working memory (beta = − 0.05, P = 2,45E-3) [53]. The same direction of results is reported for educational attainment (rg = − 0.55, P = 1,42E-151), which provides further support to the relationship between ADHD and impairments in cognitive and executive functioning [53, 60]. On the other hand, ADHD presents positive genetic correlations with other psychiatric disorders (e.g., major depressive disorder (rg = 0.31 to 0.52, P = 2,96E-06 to 2,18E-20), autism spectrum disorder (rg = 0.42, P = 1,19E-18), bipolar disorder (rg = 0.26, P = 7E-6), and schizophrenia (rg = 0.17 to 0.22, P = 1,72E-09 to 1,22E-06) and with other psychiatric-related conditions, such as neuroticism (rg = 0.23 to 0.33, P = 0.0008 to 2,61E-13), symptoms of depression (rg = 0.39 to 0.53, P = 1,25E-6 to 1,63E-32), insomnia (rg = 0.46, p = 3,73E-20), smoking initiation (rg = 0.48, P = 1,78E-11) and cannabis use (rg = 0.29 to 0.61, P = 1,63E-5 to 1,27E-42) [53, 59, 61]. Interestingly, ADHD also presents positive genetic correlations with metabolic and immune-related diseases such as overweight/obesity (rg = 0.21 to 0.27, P = 7,5E-7 to 5,46E-20), type 2 diabetes (rg = 0.18, P = 1,14E-5), asthma (rg = 0.12, P = 0,011), and psoriasis (rg = 0.23, P = 1,0E − 3) [53, 62], suggesting the possibility of a shared genetic etiology between these phenotypes. Notably, there are no genetic correlations between ADHD and most neurological diseases, such as Alzheimer’s [53, 59, 63, 64]. Even though these phenotypes are not genetically correlated, it is important to notice that there are traits, such as intelligence and cognitive performance, presenting the same direction of association to both ADHD and Alzheimer’s disease, suggesting the possibility of shared traits that contribute to both phenotypes.

The genomic studies of ADHD also reinforce the high polygenic nature of ADHD and the dose-dependent effect of the PGS for ADHD, where the increase in PGS deciles was associated with a corresponding increase in the odds ratio for ADHD in the target samples [52]. Besides the association with ADHD diagnosis, PGS for ADHD is associated with quantitative symptom measures of inattention and hyperactivity/impulsivity (even in the general population), reinforcing the view that ADHD is one extreme of a symptom distribution curve [65–67].

Relevant findings from genome-wide studies have also helped to disentangle the clinical heterogeneity of ADHD observed in different stages of life and provided insights into the debate about the neurodevelopmental conceptualization of the disorder. A GWAS comparing the genetic background between children and adults showed a shared genetic contribution to ADHD in these groups, supporting the neurodevelopmental nature of persistent ADHD in adults [68]. A subsequent study found a similar level of genetic correlation between childhood ADHD and persistent ADHD (rg = 0.82), but lower with late-diagnosed ADHD (rg = 0.65), suggesting this group might present some differences in its genetic architecture when compared to childhood ADHD [69]. Although similar patterns of cross-trait genetic correlations for children and adults were reported by Rovira et al., 2020 [68], Rajagopal et al., 2022 [69] found different patterns across ADHD subgroups for some traits, including a higher genetic correlation for childhood ADHD with autism than late-diagnosed ADHD, and for depression and alcohol use disorders for late-diagnosed ADHD compared to childhood ADHD. This suggests possible differential genetic susceptibility for common comorbidities across ADHD subgroups.

### Mendelian randomization

Despite the great advances in understanding the biological aspects related to ADHD, as summarized above, the causal effects underlying the reported associations are still largely unknown. In this regard, Mendelian Randomization (MR) has been used as a promising approach to investigate potential causal relationships between an exposure (e.g., brain measures or environmental exposures) and an outcome (e.g., ADHD susceptibility) by using genetic variants as instrumental variables. Since genetic variants are randomly determined at conception, using them as instruments has the potential to eliminate confounding effects behind associations while evaluating causal effects between a risk factor and a clinical phenotype [70, 71].

Several associations with ADHD have been explored through MR. Concerning the links with other psychiatric phenotypes, it has been suggested a causal effect of ADHD genetic liability with an increased risk for posttraumatic stress disorder [72], autism spectrum disorder (potentially a bidirectional effect [73, 74]), major depression [75, 76], substance use disorder [77, 78], including smoking [76, 78, 79], cannabis use [61, 78], and a weaker effect on alcohol dependence [78]. On the other hand, genetics of neurocognitive, such as general intelligence [80–82], and brain features (e.g. total brain volume, functional connectivity between the default mode and salience networks [80], and white matter microstructure of anterior limb of internal capsule [83] seem to represent a relevant causal role in ADHD pathogenesis, distinguishing important brain aspects playing etiological roles from those that are simply correlated with or consequences of ADHD. A bidirectional causality has also been observed between ADHD and obesity-related traits [84–86]. These findings help to disentangle the previous associations observed in cross-sectional studies, providing insights into the biological mechanisms underlying ADHD pathophysiology that can be useful for the research on potential biomarkers and preventive strategies for ADHD.

Still, the limitations of this approach should be considered while interpreting the results. MR relies on major assumptions that when violated can introduce bias to the analyses, especially regarding the absence of horizontal pleiotropy and potential confounders affecting the exposure-outcome relationship [87]. This is especially challenging for psychiatric disorders, considering their highly complex and polygenic architecture, and the involvement of pleiotropic genetic variation in their etiology. Nevertheless, alternative methodologies have been proposed to overcome these limitations, improving the interpretability and reliability of the findings, such as Mendelian Randomization Pleiotropy RESidual Sum and Outlier (MR-PRESSO) [88] and Causal Analysis Using Summary Effect estimates (CAUSE) [89].

## Neurotransmission alterations in ADHD

Despite evidence demonstrating high polygenicity in ADHD, it is possible that many variants at different loci may converge on common mechanisms. A putative pathophysiological mechanism that has been long implicated in ADHD involves the dysregulation of monoaminergic neurotransmission systems, mainly dopaminergic and noradrenergic. These neurotransmitters exert their functions by a mechanism with a curve pattern in an ‘inverted U’ shape, that is, both very intense activity (for example, during stressful situations) and very low activity (for example, states of somnolence) impair the functioning of these systems [90].

The physiological changes induced by the binding of dopamine and norepinephrine to their respective receptors involve the modulation of several cognitive and executive processes usually impaired in ADHD (see Table 1), corroborating the monoaminergic hypothesis for ADHD pathophysiology [91]. For example, dopamine receptors of subtypes D1 and D2 are abundant in brain regions mainly involved in signaling reward circuits, learning and memory, and locomotor activity [90]. Also, patients with ADHD have a higher density of the dopamine transporter (DAT), responsible for the reuptake of DA into presynaptic neurons [92], which could lead to alterations in dopamine levels in the synaptic cleft. The binding of norepinephrine with adrenergic receptors has been shown to modulate working memory processes, for which moderate or high levels of this neurotransmitter present differential binding affinities to each type of receptor and consequently different physiological effects on working memory [93]. Furthermore, the effects of methylphenidate treatment on working memory appear to be dependent on α2 noradrenergic receptors, whereas the improvement in sustained attention involves the α1 subtype [94].

**Table 1:** Neurotransmission systems with potential involvement in the pathophysiology of ADHD

Neurotransmission system	Receptors	ADHD-related functions	Evidence linking the system with ADHD^a^
Dopaminergic	D1, D2, D3, D4, and D5 dopaminergic receptors	Mediation of cognitive, motor, attentional, emotional, and reward processes [91, 99]	Higher density of the dopamine transporter (DAT) [92] and altered D2/D3 receptor availability—responsive to methylphenidate—[100–103] reported in ADHD patients. Drugs used for ADHD treatment (e.g., methylphenidate, amphetamines [104, 105], and bupropion [106]), inhibit the reuptake of dopamine
Noradrenergic	Alpha (α) and beta (β) adrenergic receptors	Regulation of cognitive functions (e.g. working memory), arousal, and alertness [91, 107]	ADHD is associated with decreased noradrenaline transporter (NET) availability in fronto-parietal-thalamic-cerebellar regions [103]. Poor performance in sustained attention tasks correlated with lower levels of urinary excretion of noradrenaline metabolites in ADHD patients [108]. Methylphenidate, amphetamines [104, 105], and atomoxetine [109] inhibit NET, while α-receptors are targeted by guanfacine and clonidine [110]
Serotonergic	5HT receptors	Involvement in sleep, appetite, mood, and emotional regulation [111]	Low serotonin levels in fasting venous blood are associated with ADHD [95]. Cerebrospinal fluid levels of serotonin positively correlated with symptoms of hyperactivity [112]. Some antidepressants acting on the serotonergic system are considered second-line alternatives for treating ADHD [113]
Glutamatergic	Ionotropic (NMDA, AMPA and kainate) and metabotropic (mGluR1-mGluR8) glutamate receptors	Regulation of executive functions (e.g., learning and memory) [114]	Increased glutamatergic tone in the frontal and striatal brain and increased glutamate levels in the anterior cingulate cortex are associated with ADHD (enhanced excitability) [96, 114]
GABAergic	GABAA and GABAB	Involvement in motor control and behavioral inhibition [115]	ADHD is associated with reduced GABA levels in the anterior cingulate, somatosensory, and motor cortices in ADHD (impaired response inhibition) [97, 116, 117]

^a^The summarized evidence presented here is restricted to pharmacological and human neuroanatomic and physiological studies

*ADHD* attention-deficit/hyperactivity disorder, *NMDA* N -methyl-D-aspartate, *AMPA* α-amino-3-hydroxy-5-methyl-4-isoxazole-propionic acid, *GABA* gamma-aminobutyric acid, *5HT* serotonin

Although not as extensively as the dopaminergic and noradrenergic systems, the involvement of serotonergic [95], glutamatergic [96], and GABAergic [97] systems have also been implicated in ADHD by studies associating altered levels of these neurotransmitters with ADHD (Table 1). Experimental models also demonstrated that methylphenidate is able to restore impaired glutamatergic transmission at the prefrontal cortex and improve behavioral symptoms of ADHD, a process that appears to be mediated by AMPA glutamatergic receptors [98].

Besides the above-mentioned evidence, the involvement of neurotransmitter systems in ADHD is reinforced by the mechanism of action of most drugs used for its treatment that involves targets related to these biological pathways (e.g., methylphenidate, which targets DAT and NET). Additional evidence for the involvement of these neurotransmission systems in ADHD is summarized in Table 1. Nevertheless, ADHD involves a complex neurobiology that results from the interaction between various dysfunctional neurophysiological systems, as evidenced by genomic studies.

## Brain structural and functional alterations in ADHD

Pathophysiological insights can also be inferred from neuroimaging studies, which has reported differences in structural and functional brain architecture between patients with ADHD and neurotypical individuals, especially in children. Large meta-analyses from the ADHD working group of the ENIGMA (Enhancing NeuroImaging Genetics through Meta-Analysis) consortium reported that children with ADHD present smaller volumes in different subcortical brain regions (i.e., nucleus accumbens, amygdala, caudate, hippocampus, and putamen) and total intracranial volume [118], as well as decreased cortical surface area (mainly in frontal, cingulate, and temporal regions) and thickness (in the fusiform gyrus and temporal pole) [119]. Such alterations are not observed when restricting the analyses to adults and no effects of psychostimulants were found [118, 119]. Through adopting an independent component analysis of whole-brain morphometry images, associations of ADHD with reduced volumes in frontal lobes, striatum, and their interconnecting white matter microstructure were reported for both children and adults, supporting a role of the frontostriatal circuit regardless of age [120]. A large cross-sectional brain imaging study, the Adolescent Brain and Cognitive Development (ABCD), also suggests reduced structural brain measures in children with ADHD, although with modest effect sizes [121], indicating additional investigation is needed to support specific cortical and subcortical alterations as potential biomarkers for ADHD.

Structural connectivity differences in ADHD have been investigated using Diffusion Tensor Imaging (DTI), a technique based on the observation of the directionality and coherence of water diffusion that allows inferences on the microstructure of brain white matter (WM). Meta-analyses based on fractional anisotropy (FA) measures, a common DTI parameter that is considered a marker of white matter microstructural integrity, support that the most robust findings related to ADHD involve the frontostriatal circuitry and the interhemispheric connection through the corpus callosum [122, 123]. Microstructural alterations in the cingulum and corticospinal tracts are also commonly associated with ADHD. However, contrasting DTI findings have been reported, with either reduced or increased FA measures being observed for the same brain circuitry. These disparities are observed for different methodological approaches, for example, voxelwise or tractography-based, and might reflect potential confounders as sources of heterogeneity among studies, such as non-controlled head motion group differences [122]. A recent large multi-cohort mega-analysis that used original DTI data attempting to overcome these issues showed that lower FA of inferior longitudinal (ILF) and left uncinate (UNC) fasciculi were associated with both ADHD diagnosis and symptoms (attention problem scores) [124]. These findings are consistent with a pathophysiological mechanism for ADHD that involves brain networks related to cognitive functions (ILF) and emotion/reward processing (UNC). Although the general landscape for the role of the implicated regions is congruent with functions usually impaired in ADHD, such as cognition, behavioral plasticity, and emotional and motor control, larger and more homogeneous studies are necessary to validate them as key factors in the neurobiology of ADHD.

Functional Magnetic Resonance Imaging (fMRI) studies associate ADHD with alterations in connectivity in multiple brain networks involved mainly in executive functions, attention, emotions, and sensory and motor activities. A broadly studied model for ADHD suggests an impaired synchronization between the DMN (Default Mode Network), which is normally more active when tasks are not performed, and the TPN (Task-Positive Network), which is activated while performing tasks. In individuals with ADHD, inadequate DMN hyperactivity and TPN hypoactivity while performing tasks have been suggested as an explanation for ADHD symptoms [125]. In resting-state fMRI (rsfMRI) studies, the frontoparietal network, involved in executive functions, has demonstrated hyperconnectivity with regions of the DNM and affective network, while hypoactivity with regions of the ventral attention and somatosensory networks in individuals with ADHD [126]. ADHD has also been associated with disrupted connectivity between DNM and systems involved in cognitive control and affective/motivational and salience networks [127]. A large mega-analysis of rsfMRI supports the findings suggesting that ADHD is associated with stronger coactivation/weaker anticorrelation between DNM and TPN networks, including the dorsal and salience/ventral attention networks, which is thought to result in attention lapses/mind-wandering and worse performance on attention and executive function tasks [128]. Impaired functional connectivity in these networks has been supported by task-based studies across several cognitive domains [129]. Congruently, methylphenidate treatment is associated with the suppression of DNM activity during cognitive tasks and the improvement of resting state functional connectivity within DMN in patients with ADHD [130, 131]. However, a recent meta-analysis of rsfMRI reported no significant spatial convergence of hyperconnectivity or hypoconnectivity related to ADHD across studies [132]. These results reflect once again the high heterogeneity of neuroimaging studies, including differences in experimental methods, characteristics of participants, and analytic design, as well as the heterogeneous presentation of ADHD and its complex pathophysiology.

Although methodological issues challenge the identification of brain aspects related to ADHD, such associations are supported by genomic studies showing a high genetic correlation between ADHD and several neuroimaging measures, including intracranial and total brain volumes and cortical surface area [133–136]. Additionally, it was suggested the neuroimaging associations observed in children may also be detected in adults depending on the genotype profile [137]. Therefore, the genetic architecture of ADHD may be shared to some extent with brain structure variation in both children and adults.

## Epigenomics

Some studies evaluating differential methylation in candidate genes were conducted in ADHD [43, 138]. However, as occurred for GWAS, the number of epigenome-wide association studies (EWAS) is growing. EWAS aims to identify epigenetic modifications involved in the susceptibility and course of phenotypes in a large-scale analysis, which evaluates methylation sites spread throughout the entire genome. In the case of ADHD, at least 11 EWASs have already been published, two of which are meta-analyses [139–149]. Unfortunately, several single-site EWAS did not reveal positions (differentially methylated positions—DMPs) in the epigenome-wide significance level. However, some promising results can already be summarized, mainly from analyzes involving differentially methylated regions (DMRs). For example, three studies identified *VIPR2* (Vasoactive Intestinal Peptide Receptor 2) as a differentially methylated gene in ADHD [139, 140, 147]. In addition, one study demonstrated differential methylation in the *ST3GAL3* gene (ST3 Beta-Galactoside Alpha-2,3-Sialyltransferase 3) in the epigenome-wide significance level [143], which was also associated with ADHD by GWASs [52, 53].

The two meta-analyses involved different study designs [145, 148]. One meta-EWAS included only children and adolescents and looked at ADHD trajectories. Significant genes in the case–control analysis involving cord blood (n = 2477) were not implicated in ADHD symptomatology during childhood and adolescence (n = 2374). At birth, nine CpGs predicted later ADHD symptoms in genes that are not GWAS hits for psychiatric disorders yet (*ERC2*—ELKS/RAB6-interacting/CAST family member; *CREB5*—cAMP responsive element binding protein 5; *ZBTB38*—zinc finger and BTB domain containing 38; *PPIL1*—peptidylprolyl isomerase like 1; and *TRERF1*—transcriptional regulating factor 1) [148]. The second one [145] was a meta-EWAS of ADHD symptoms in adults that included three individual cohorts (the Netherlands Twin Register, Dunedin Multidisciplinary Health and Development Study, and Environmental Risk Longitudinal Twin Study). The combined sample size was 4,689 individuals. One epigenome-wide significant DMP was detected in the Dunedin study (chromosome 8 intergenic), but this finding was not observed in the meta-EWAS of the three cohorts. In region-based analyses, six DMRs were identified in the Netherlands Twin Register, 19 in the Dunedin study, and none in the Environmental Risk Longitudinal Twin Study. No significant DMR was associated with more than one cohort. The top single-cohort gene was *TNXB*, a genome-wide hit in the schizophrenia and neuroticism GWASs [150–152].

Although the results summarized here are promising, this set of studies is still small, especially involving samples of adult individuals and looking at clinical aspects of ADHD (only three EWASs). Further studies with even larger samples are needed to elucidate the role of epigenetic mechanisms in ADHD throughout life. Additionally, the relationship between epigenomics and genomics should be better studied since epigenetic markers also present significant heritability estimates [153]. A recent study evaluating global DNA methylation (GMe) showed that the GMe levels observed in ADHD cases were lower than controls. Furthermore, the ADHD PGS was negatively correlated with GMe in ADHD cases [154]. On a locus-specific level, the ADHD PGS was associated with differential methylation in an epigenome-wide significant level with two genes that were not implicated in ADHD or other psychiatric disorders by GWASs yet (*GART*—phosphoribosylglycinamide formyltransferase, phosphoribosylglycinamide synthetase, phosphoribosylaminoimidazole synthetase, and *SON*—DNA and RNA binding protein) [147]. Therefore, these findings demonstrated a degree of genetic control over methylation driven by the underlying genomics of ADHD, which should be further explored as a potential mechanism for gene-environment interactions.

## Metagenomics

The bidirectional communication between the gut microbiota and the nervous system is known as the gut-brain axis (GBA). The main mechanisms through which the gut microbiota could affect the nervous system involve the production and metabolism of neurotransmitters, the vagus nerve, the hypothalamic–pituitary–adrenal axis, and inflammation pathways [155, 156]. The unbalance of microbiota composition—dysbiosis—can lead to systemic inflammation, dysregulation of neurotransmitter levels, and increased oxidative stress, affecting brain functions via the gut-brain axis.

The role of microbiota in the pathogenesis of ADHD has been suggested by studies showing differences in microbiota composition and/or abundance between patients with ADHD and controls [157]. An increased abundance of *Enterococcus*, *Bifidobacterium*, and *Odoribacter* has been reported in ADHD patients. These genera are involved in pathways related to the production and release of the neurotransmitters, such as GABA and dopamine, suggesting that such gut dysbiosis could be related to the pathogenesis of ADHD through neurotransmitter dysregulation [158–160]. On the other hand, the abundance of *Faecalibacterium* is decreased in ADHD and is inversely related to ADHD severity [159, 161, 162]. *Faecalibacterium* has been related to anti-inflammatory properties [163]. Unbalanced levels of this genus could influence the production of pro-inflammatory cytokines and induce systemic inflammation, a mechanism that has been proposed to contribute to ADHD pathogenesis [164, 165].

The specific differences in gut microbiota mentioned above describe the most common findings that have been highlighted by recent reviews; however, a meta-analysis that assessed the relationship between gut microbiota and ADHD found no significant differences at the phylum and family levels, reporting as the only significant association the higher within-group diversity of *Blautia* in patients with ADHD compared to healthy controls [166]. *Blautia* is known to interfere with the regulation and function of inflammation and immunity pathways, reinforcing the hypothesis that the link between gut microbiota and ADHD pathogenesis might involve inflammation mechanisms.

Regarding the general composition of the microbiota, while some individual studies have found differences in patients with ADHD in diversity indices (alpha and beta) [167, 168], the overall evidence and meta-analyses point to high heterogeneity across studies and do not support a consistent association of diversity indices with ADHD [160, 166]. In general, the studies evaluating the microbiota effects on ADHD are limited, and contradictory/inconsistent findings have been reported, likely due to methodological differences and small sample sizes [157].

## ADHD treatment

Early and adequate intervention has the potential to reduce the risk of negative ADHD-related outcomes in mental and physical health [169]. The treatment options can be non-pharmacological, pharmacological, or a combination of both. The first comprises psychosocial, cognitive, and behavioral training that stimulates specific neuropsychological domains associated with ADHD, such as cognitive and executive functions. According to meta-analyses, patients may experience some benefits of such psychotherapies compared to placebo, and they are especially recommended as first-line or adjunct treatment for young children (aged 6 years or less) and less severe cases [170–172]. However, for most of these psychotherapeutic interventions, meta-analyses point to low certainty and inferior efficacy in reducing the core symptomatology of ADHD compared to the pharmacological treatment (e.g., with stimulants) [170].

Therapeutic guidelines, including those from the British Association of Psychopharmacology [173] and the National Institute for Health and Care Excellence [171], generally recommend stimulant medications, namely lisdexamfetamine and methylphenidate, as the first-choice pharmacological treatment for moderate to severe ADHD cases and patients aged 6 years or older. For patients who neither tolerate nor respond to treatment with these drugs, non-stimulants (e.g., atomoxetine) are considered the second choice, followed by adrenergic agents (e.g., clonidine and guanfacine) or alternative non-stimulant drugs, including antidepressants such as tricyclics and bupropion [171, 173–175]. As detailed below, the intervention options have varying properties in terms of effect size, mechanism of action, as well as different profiles of side effects. The treatment strategy depends on these factors and should be carefully evaluated on an individual basis according to the clinical profile of the patients, especially considering the presence of comorbidities, which are frequently observed in patients with ADHD.

### ADHD medications

The safety and efficacy of commonly used ADHD medications are supported by metanalyses (Cortese et al., 2018), which provide the basis for the guidelines’ recommendations mentioned above. Besides being efficacious in reducing the core symptomatology of ADHD, there is congruent evidence indicating their potential to improve related outcomes, such as quality of life, academic performance, social aspects, comorbid neuropsychiatric conditions, as well as reduce the risk of other functional impairments, including criminality, incidents of self-harm, suicidality, and risk-taking behaviors [169, 176–179].

#### Stimulants

In general, stimulants present superior efficacy compared to other medications for all age ranges. Among the stimulants, amphetamines have shown greater effect sizes in reducing ADHD symptoms than methylphenidate [180]. Although the benefit of methylphenidate on ADHD symptoms is also observed in the long-term when compared to discontinuation or switching to a placebo, the long-term effect sizes are usually smaller than those reported in short-term studies of methylphenidate treatment [174].

Methylphenidate, the most used medication worldwide, is derived from piperidine and structurally similar to amphetamine. Both methylphenidate and amphetamines produce similar effects in increasing the availability of dopamine and norepinephrine in the synaptic cleft, leading to enhanced neurotransmission, but their mechanisms of action differ in some respects. The pharmacological activity of methylphenidate results mainly from blocking the dopamine and norepinephrine transporters (DAT and NET, respectively), thus inhibiting the reuptake of these neurotransmitters into presynaptic neurons [104]. On the other hand, besides the inhibition of DAT and NET through the direct binding to them as a false substrate, amphetamines also promote increased presynaptic efflux of dopamine into the extracellular space. The regulation of this process involves the modulation of functions such as attention, pleasure, and motor activity [105]. Stimulants also improve executive functions frequently impaired in individuals with ADHD, such as inhibitory control, working memory, and sustained attention in an age-independent manner [181–183].

Amphetamines and methylphenidate present with a similar profile of adverse effects, which are usually mild and transient. The most common are decreased appetite, dry mouth, irritability, sleep disturbances, tachycardia, and headache; however, amphetamines are associated with a higher rate of side effects, such as weight loss and insomnia [170]. Concerns about stimulant use and adverse cardiovascular outcomes, including increases in heart rate or blood pressure, have also been raised [180]. However, the relationship between these medications and severe cardiovascular events has been controversially discussed since large registry studies do not support a clear association between serious cardiovascular outcomes and stimulant use [184, 185]. Additional caution should be taken when using stimulants to treat patients with ADHD in comorbidity with substance use disorders because of the addictive potential of these medications, especially the short-acting formulations [186]. In these cases, alternative non-stimulant treatment options or long-acting stimulant formulations are most suitable [187]. Treatment of ADHD with comorbid bipolar disorder also requires special care because of the risk of stimulants inducing mixed/manic episodes in these patients [188, 189].

#### Non-stimulants

For patients who do not respond, do not tolerate, or present specific conditions for which stimulants are contraindicated, the non-stimulant medications atomoxetine, clonidine, and guanfacine are alternative FDA-approved treatment options. Meta-analyses confirm that all these medications are safe and superior to placebo in reducing ADHD symptoms, and the specificities of each drug are described below.

In clinical assessments, atomoxetine presents efficacy of modest effect size [180] but is still superior to placebo in reducing the severity of ADHD symptoms. Compared to stimulants, atomoxetine is less effective and usually has a slower initial response, with symptom improvement being noticed only after several weeks of treatment. Besides, a considerable percentage (~ 40%) of patients persist with impairing symptoms while being treated with atomoxetine, demanding additional management [109]. Nevertheless, atomoxetine might be particularly indicated in cases of comorbid ADHD and anxiety or for patients with a history of substance abuse, for which it may be considered the first treatment option [190, 191]. Regarding tolerability, the most observed adverse events include nausea, loss of appetite, fatigue, dizziness, irritability, abdominal pain, and sleep disturbances [109]. Considering the potential risk of suicidal behavior reported by clinical trials in children receiving atomoxetine, although not supported by meta-analyses [192], it is recommended to monitor closely any notable behavior shift mainly at the beginning of treatment or dose adjustment in children.

Atomoxetine is a potent and selective inhibitor of norepinephrine reuptake that acts through the blockade of the NET. Such presynaptic NET inhibition leads to increased norepinephrine extracellular levels and occurs mainly in the prefrontal cortex but not in the mesolimbic and mesocortical pathways up to the nucleus accumbens. Therefore, atomoxetine presents minimal abuse or dependence potential [191]. Although atomoxetine presents a low affinity for DAT, it also promotes increased dopamine concentrations in the prefrontal cortex, which may result from the nonspecific modulation of dopamine reuptake via NET, which is highly expressed in this brain region. Atomoxetine has little or no affinity for noradrenergic receptors or any other type of receptor [193].

Extended-release formulations of the α2-adrenergic agonists clonidine and guanfacine are also second-choice alternatives for ADHD treatment, both as monotherapy and as an adjunct to stimulants. Although monotherapy with these agents has shown superior efficacy to placebo, when compared to stimulant medications, they have smaller effect sizes and their treatment effects take longer to be observed [110, 180, 194, 195]. However, the administration of these drugs as an add-on therapy to stimulant medications has been recommended since the combined mechanism of action of both drug classes may result in clinical improvement in ADHD symptomatology or a decrease in adverse effects [110, 196].

Guanfacine and clonidine have similar pharmacological properties and, differently from atomoxetine, their actions to potentiate the noradrenergic transmission occur through the direct agonism of the postsynaptic α2-adrenergic receptors rather than via NET blockade [196]. However, they differ in terms of potency and affinity. Guanfacine is a much less potent agonist than clonidine and seems to have a higher affinity for α2A receptors, while clonidine binds less selectively to all three subtypes of α2 receptors. In addition, differently from guanfacine, clonidine has the potential to act on imidazoline receptors as well, which would explain the more pronounced sedative and hypotensive effects of clonidine [110, 197]. Common adverse effects of both clonidine and guanfacine include somnolence/sedation, fatigue, irritability, insomnia, and nightmares, and they are usually more frequent and more pronounced than those observed for atomoxetine [195].

#### Other therapeutic alternatives

Although not included in clinical guidelines, therapeutic alternatives, such as the antidepressant bupropion, have also been used in clinical practice to treat ADHD symptoms. In this sense, there is evidence of its effectiveness, especially at high doses, although the consistency across studies is not too strong [198, 199]. The prescription of this medication might be specially considered as an option when comorbid conditions such as mood disorders or nicotine dependence are present, considering its primary antidepressant effect and its contribution to smoking cessation [200, 201]. Bupropion, unlike other typical antidepressants, such as tricyclics, promotes the inhibition of dopamine and norepinephrine reuptake only, and not serotonin. This drug also acts as a non-competitive antagonist of nicotinic acetylcholine receptors. Its mechanism of action is similar to that of stimulants, but the inhibition of neurotransmitter reuptake induced by bupropion is weaker than that produced by stimulants [199].

### Pharmacogenomics

Although the approved medications are generally safe, efficacious, and lead to an important relief of ADHD core symptoms, they are not curative, and a considerable proportion of patients do not reach an optimal response. Besides, their effectiveness is further reduced due to high rates of poor drug adherence and persistence [202]. Such heterogeneity in treatment response, which may reflect the clinical heterogeneity of ADHD itself, affects treatment decision-making and challenges the definition of concise recommendations to guide clinicians. Thus, addressing the risks and benefits of the available medications for ADHD, the possibility of drug repurposing of approved compounds and the development of new therapeutic alternatives are the focus of current research to improve the treatment outcomes of patients with ADHD.

As briefly reviewed above, the known pharmacological actions of common medications used to treat ADHD involve only a few biological pathways, which seems inconsistent with the high complexity of the pathophysiological mechanisms proposed for ADHD. In this sense, GWAS arises as an important tool to advance knowledge for a more comprehensive understanding of the molecular underpinnings of ADHD neurobiology and the medications used for its treatment, helping to guide the development of better strategies for patient care [203].

As GWAS of ADHD susceptibility increases in power, they can reveal additional potential pathways involved with the disorder’s pathophysiology and yield new candidates for prospective drug development. Nonetheless, no significant genome-wide results have been found in GWAS for the response to treatment in patients with ADHD [204, 205]. The lack of success can be explained by the small sample sizes with genomic and treatment data available worldwide. However, promising insights can be inferred by applying strategies such as polygenic risk scores to test the genetic overlap between ADHD treatment response and the genetic liability for ADHD or other psychiatric traits. For example, higher polygenic risk scores for ADHD were associated with symptomatic improvement following ADHD medication in a small study using a Chinese sample [206], suggesting shared molecular mechanisms between ADHD and treatment response.

On the other hand, in a study investigating the druggable genome, the genes targeted by the FDA-approved medications for ADHD were not associated with ADHD at the genome-wide level, indicating that these drugs act through alternative pathways different from those underlying ADHD neurobiology [207]. Another interesting finding from the Lundbeck Foundation Initiative for Integrative Psychiatric Research (iPSYCH2012) case-cohort [208] suggests that ADHD cases with higher polygenic liability for mood and/or psychotic disorders might be at increased risk of discontinuing or switching stimulant treatment. Other studies applying alternative strategies taking advantage of integrative genome-wide, expression, and proteomic data from experimental studies have also reported some insights into the genetic background possibly involved with treatment response. They have revealed pathways involved in nervous system development and function, neurotransmitter release, GABA transmission, as well as other categories related to neurological diseases, psychological disorders, and behavior [205, 209]. Interestingly, they did not point specifically to the pathways largely implicated in ADHD, namely the dopaminergic and/or noradrenergic transmission, suggesting that additional mechanisms might underlie ADHD neurobiology and drug actions.

Although the evidence from candidate gene studies suggests a genetic contribution related to the dopaminergic and noradrenergic pathways in the treatment response of ADHD, especially in children, few robust and replicated results have been observed across these studies. A meta-analysis conducted by Myer et al. (2017) suggests polymorphisms in the *SLC6A2*/*NET*, *COMT*, *ADRA2A*, *SLC6A3*/*DAT1* and *DRD4* genes as potential predictors of the efficacy of methylphenidate in children [210]. For adults, a meta-analysis carried out by Bonvicini et al. (2016) concluded that for most polymorphisms, there is not enough data to apply this methodology. The only polymorphism for which it was possible to perform the meta-analysis was the 40-base pair VNTR in the *DAT1* gene, for which no significant association with the response to methylphenidate was found [54].

The current limited knowledge provided by genetic studies of ADHD treatment so far reinforces the need to improve the understanding of the molecular basis of ADHD treatments. As further insights on the molecular biology of ADHD and medication used to treat it emerge, advancements in the pharmacotherapeutic options for ADHD improve, potentially benefiting the care of patients with ADHD. The above-mentioned exploration of the druggable genome in ADHD based on GWAS yielded processes of signal transduction and cell adhesion as novel possibilities for intervention in ADHD treatment, presenting possibilities of drug repurposing and novel targets for drug development [207]. A potentially prosperous example of drug repurposing based on molecular knowledge is the use of fasoracetam monohydrate, originally tested in clinical trials for vascular dementia, to treat ADHD. Fasoracetam is a metabotropic glutamate receptor activator and a clinical trial demonstrated it is efficacious in reducing symptoms of ADHD in cases of impaired glutamatergic signaling, a common pathway implicated in ADHD by genetic associations studies [211].

## Concluding remarks

ADHD is an important impairing condition of public health due to its prevalence and persistence across the lifespan, and because it leads to a higher risk of adverse outcomes, such as academic underachievement, substance use and abuse, other psychiatric disorders, somatic diseases, risky behaviors, and premature death. Although the existing medications are considered safe and efficacious in relieving symptoms and preventing many negative consequences, there are groups of individuals with inappropriate responses, and these drugs’ long-term therapeutic effects are not robust. Research aiming to provide a more comprehensive understanding of the disorder’s pathophysiology is warranted to improve the diagnosis and management of the disorder.

**Fig. 1:**
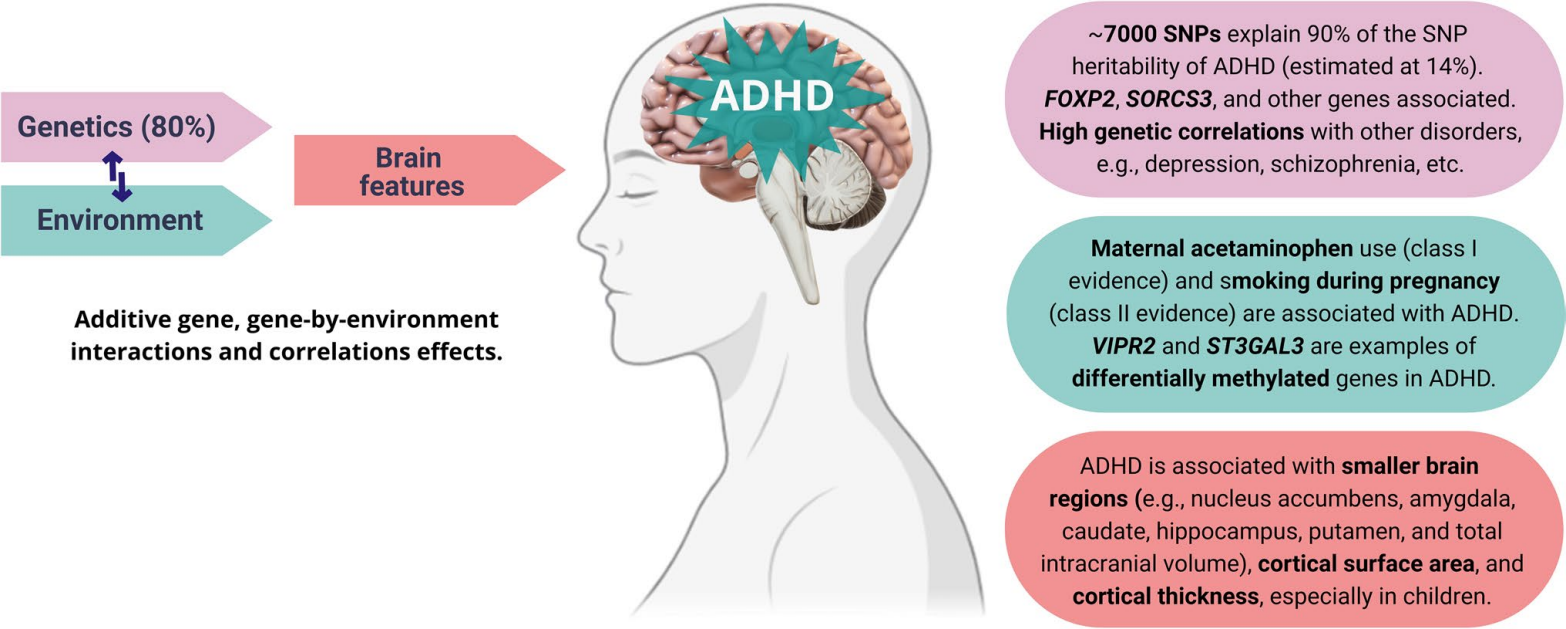
Multifactorial complexity of Attention-Deficit/Hyperactivity Disorder (ADHD). ADHD presents substantial heritability in children, adolescents, and adults (the polled average is nearly 80%). Genetic and environmental factors modulate brain features (e.g., regional volumes, cortical thickness, connectivity, etc.) that are potentially in the causal pathway of the disorder

Hundreds of studies have attempted to identify the underlying neurobiology or biomarkers for ADHD. These studies have faced several challenges, especially those related to the high heterogeneity of the disorder. Regardless, there is no doubt about the role of biology in the etiology and course of ADHD, and some facts are already a consensus in the area [7] (Fig. 1). For example, ADHD is substantially influenced by genetics with a polygenic architecture, there is extensive biological and phenotypic overlap between ADHD and other psychiatric traits, and the correlations observed in clinical and epidemiological studies have been confirmed by studies of genetic correlations. However, it is still necessary to increase sample sizes through large international collaborations to reveal additional genetic loci involved in ADHD susceptibility, increase the accuracy of polygenic scores, and ultimately understand the biological meaning of the findings and their utility in clinical practice.

The study of brain biology has been somehow boosted by the development of neuroimaging techniques. The combined effects of risk factors may affect brain regions and networks that control cognitive, executive, and emotional processes usually impaired in ADHD, which can be evaluated through neuroimaging. These studies have helped to understand the functional and structural aspects of ADHD and added to our understanding of which brain areas are modulated and/or modified during the pharmacological treatment of ADHD. Despite the growing progress, the findings should be interpreted with caution due to the small sample sizes evaluated in these studies added to the high heterogeneity of both neuroimaging study designs and ADHD presentation.

The role of the environment in ADHD has also been the focus of several studies. Still, the reported associations should be viewed as correlated rather than causal risk factors. Since both environment and genetics interact in a complex manner in the contribution to the etiology of ADHD and the high probability of unmeasured factors in the studies, it is a challenge to identify environmental risk factors robustly associated with ADHD without the interference of confounders. Epigenomics and metagenomics will certainly become important tools in studying the role of the environment in the etiology and course of ADHD. While these approaches are promising, the available sample sizes are still very small. From this perspective, international consortia will be essential to meet this need for larger sample sizes. An important example is Eat2BeNice, a multicentric and international project whose main goal is to investigate the effects of nutrition and lifestyle on ADHD using genomics, epigenomics, and metagenomics as research tools (https://newbrainnutrition.com).

It is also a consensus that pharmacogenomics, at this first moment, will be useful for understanding the biological mechanisms involved in the therapeutic response rather than providing cost-effective tests. The application of pharmacogenetic tests is still far from benefiting clinical practice. While advancing the knowledge in ADHD neurobiology and treatment response, the development or repurposing of drugs with different targets from those of the current medications is pursued to improve patient care.

## Future directions

Considering ADHD dimensionality and multifactorial etiology, several measures have been related to ADHD and its treatment, including clinical symptomatology, cognitive functioning, environmental factors, brain aspects, and biological signatures (Fig. 1). However, no unique measure is proven to guide diagnosis or treatment strategies. Promising biomarkers will probably combine numerous domains of measurement.

Although the knowledge provided by epidemiological and biological studies has significantly improved in the last few years, it is still a long way to comprehensively understand the causes and disentangle all the heterogeneity of this disorder. It is necessary to increase sample sizes to improve the power of genomic studies, which will also allow investigating the genetic influence on the trajectories of the disorder, helping to elucidate, for example, the occurrence of late-onset ADHD that has been demonstrated in population studies, and other sources of heterogeneity. In addition, most studies have focused on European ancestry samples, and extrapolating the findings to other ethnicities is not guaranteed. Future research should investigate more diverse samples and consider diverse cultural contexts. In this sense, international collaborations such as the Latin American Genomics Consortium (LAGC) are important initiatives to advance psychiatric genetics research in populations other than European.

The growing body of research and technological advances provide good perspectives for understanding the neurobiology of ADHD, refining diagnosis, and identifying new therapeutic options to optimize treatment outcomes and associated impairments, leading to improvements in all domains of patient care.

## Supplementary Information

Below is the link to the electronic supplementary material.

**Additional file 1: Table S1.** Meta-analyses summarizing effects from exposures to several substances (prenatal and postnatal). **Table S2.** Meta-analyses summarizing associations with biomarkers and nutritional factors. **Table S3.** Meta-analyses summarizing associations with parental, newborn, and lifespan-related factors.

## Data Availability

Not applicable.
