# Supplementary material for: An overview on neurobiology and therapeutics of attention-deficit/hyperactivity disorder
Source: Discov Ment Health. 2023 Jan 5;3(1):2. doi: 10.1007/s44192-022-00030-1 (PMC10501041; doi:10.1007/s44192-022-00030-1)
Supplement: Supplementary file 1 — Additional file 1: Table S1. Meta-analyses summarizing effects from exposures to several substances (prenatal and postnatal). Table S2. Meta-analyses summarizing associations with biomarkers and nutritional factors. Table S3. Meta-analyses summarizing associations with parental, newborn, and lifespan-related factors. [file 44192_2022_30_MOESM1_ESM.docx]

**Additional information**

**Discover Mental Health**

**An overview on neurobiology and therapeutics of attention-deficit/hyperactivity disorder**

Bruna Santos da Silva^1,2,4,5^, Eugenio Horacio Grevet^1,3^, Luiza Carolina Fagundes Silva^1,3^, João Kleber Neves Ramos^4^, Diego Luiz Rovaris^4^, Claiton Henrique Dotto Bau^1,2,3^

^1^ADHD and Developmental Psychiatry Programs, Hospital de Clínicas de Porto Alegre, Universidade Federal do Rio Grande do Sul, Porto Alegre, Brazil.

^2^Department of Genetics and Graduate Program in Genetics and Molecular Biology, Instituto de Biociências, Universidade Federal do Rio Grande do Sul, Porto Alegre, Brazil;

^3^Department of Psychiatry and Graduate Program in Psychiatry and Behavioral Sciences, Faculdade de Medicina, Universidade Federal do Rio Grande do Sul, Porto Alegre, Brazil;

^4^Department of Physiology and Biophysics, Instituto de Ciencias Biomedicas Universidade de Sao Paulo, São Paulo, Brazil.

**Corresponding author:**

Claiton Henrique Dotto Bau (claiton.bau@ufrgs.br)

**Additional file 1: Table S1 Meta-analyses summarizing effects from exposures to several substances (prenatal and postnatal)**

| **Substance** | **Exposure period** | **Main finding(s)** | **N studies** | **Sample size** | **Observations** | **Ref.** |
| --- | --- | --- | --- | --- | --- | --- |
| Acetaminophen | Prenatal | Children prenatally exposed to acetaminophen were more likely to subsequently have ADHD symptoms. The associations were slightly stronger among boys. | 6 | >10k | Small heterogeneity. | Alemany et al. 2021[1] |
| Acetaminophen | Postnatal | Not significant. | 6 | >10k | Small heterogeneity. | Alemany et al. 2021[1] |
| Acetaminophen | Prenatal | Acetaminophen use during pregnancy was associated with an increased risk of ADHD. | 7 | >150k | Moderate heterogeneity. Association potentially confounded by parental ADHD. | Masarwa et al. 2020[2] |
| Acetaminophen | Prenatal | Maternal exposure during pregnancy increased the risk of ADHD. A longer duration (≥ 28 days) of acetaminophen use during pregnancy was correlated with a higher risk. | 8 | >200k | Moderate heterogeneity. A stronger association was observed in studies adjusted for child gender. | Gou et al. 2019[3] |
| Acetaminophen | Prenatal | Acetaminophen use during pregnancy was associated with an increased risk of ADHD and hyperactivity symptoms. | 6 | >100k | Moderate to high heterogeneity. Six studies included in the ADHD and five in the hyperactivity symptoms meta-analysis. | Masarwa et al. 2018[4] |
| Alcohol | Prenatal and Postnatal | Low–moderate prenatal alcohol exposure (≤20, ≤50, and ≤70 g/week) was not associated with ADHD. | 6 | >50K | Moderate to high heterogeneity. Two studies included in the ≤20, four in the ≤50, and six in the ≤70 meta-analysis. | San Martin Porter et al. 2019[5] |
| Alcohol | Prenatal | Evidence of an increased risk of ADHD in those with prenatal alcohol exposure (PAE) beyond that due to parental alcohol dependence or a genetic liability. | 16 | >10k | High heterogeneity. The risk of ADHD was higher in prenatal exposure studies compared to studies evaluating children of parents with alcohol use disorder. | Wetherill et al. 2018[6] |
| Alcohol | Prenatal | Individuals with PAE presented increased risk of internalizing and externalizing problems. The occurrence of total behavior problems was similar to that seen in ADHD samples. | 65 | >10k | High heterogeneity. Forty-one studies included in the internalizing, fifty-five in the externalizing, and six in the comparison of “alcohol-exposed and ADHD samples” meta-analysis. Several factors were found to moderate the impact of PAE on behavior outcomes. | Khoury et al. 2018[7] |
| Antibiotics | Postnatal | Not significant. | 3 | >750k | High heterogeneity. | Duong et al. 2022[8] |
| Antibiotics | Prenatal | The use of antibiotics during pregnancy was associated with ADHD. | 4 | >1M | Moderate heterogeneity. | Ai et al. 2021[9] |
| Antibiotics | Postnatal | Not associated. | 8 | >1M | High heterogeneity. The association was significant in the sensitivity analysis after the exclusion of two studies. | Ai et al. 2021[9] |
| Antibiotics | Postnatal | Antibiotic exposure was associated with an increased risk of ADHD. However, this association disappeared when data from sibling-matched studies were pooled. | 6 | >1M | High heterogeneity. Three studies included in the sibling-matched meta-analysis. Potentially explained by unmeasured genetic and familial confounding factors. | Yu et al. 2021[10] |
| Antidepressants (SSRIs) | Prenatal | There was an association between exposure during 1st trimester of pregnancy and increased risk of ADHD. | 5 | >2M | Small to moderate heterogeneity. Three studies included in the OR and two in the RR meta-analysis. Potentially biased by indication. | Halvorsen et al. 2019[11] |
| Antidepressants (SSRIs SNRIs) | Prenatal | There was an association between antidepressant exposure during pregnancy and the risk of ADHD. Preconception exposure was associated with risk of ADHD. | 6 | >1M | Moderate heterogeneity. Potentially biased by indication. | Leshem et al. 2021[12] |
| Antidepressants (SSRIs and non-SSRIs) | Prenatal | There was an association between antidepressant exposure during pregnancy and the risk of ADHD. Preconception exposure was associated with risk of ADHD. | 6 | >2M | Small to high heterogeneity. Three studies included in the preconception use meta-analysis. Potentially biased by indication. | Jiang et al. 2018[13] |
| Antidepressants (SSRIs and non-SSRIs) | Prenatal | There was an association between antidepressant exposure during pregnancy and the risk of ADHD. Preconception exposure was associated with risk of ADHD. | 7 | >2M | Small to moderate heterogeneity. Five studies included in the preconception use meta-analysis. Potentially biased by indication. Not significant in the sibling design (three studies). | Morales et al. 2018[14] |
| Antidepressants (SSRIs and non-SSRIs) | Prenatal | There was an association between antidepressant exposure during pregnancy and the risk of ADHD. Preconception exposure was associated with risk ADHD. | 7 | >2M | Small to high heterogeneity. Five studies included in the preconception use meta-analysis. Potentially biased by indication. Not significant in the sibling design (three studies). | Man et al. 2018[15] |
| Bisphenol A | Prenatal and Postnatal | Associated to increased hyperactivity in male rodents. There were only three studies carried out in humans. Therefore, they were not meta-analyzed. | 12 | >1k | Small heterogeneity. | Rochester et al. 2018[16] |
| General anesthetics | Postnatal | Single exposure to general anesthesia did not increase the risk of ADHD. Multiple exposures to general anesthesia did increase the risk of ADHD. | 4 | >25k | Small to high heterogeneity. | Song et al. 2021[17] |
| General anesthetics | Postnatal | Repeat childhood general anesthesia, but not one-off general anesthesia was associated with ADHD in later life. | 7 | >300k | Small to moderate heterogeneity. The association was evident only when the total general anesthesia exposure exceeded 90 min. Seven studies included in the “overall”, four in the “single”, five in the “two or more”, and two in the “≥ 90 minutes” meta-analysis. | Sun et al. 2021[18] |
| Lead | Postnatal | Lead exposure increased the risk of ADHD | 13 | >7k | High heterogeneity. | Nilsen and Tulve 2020[19] |
| Lead | Postnatal | Lead exposure increased ADHD symptoms even for children with low blood lead levels (<3 μL/dL). | 7 | >2k | High heterogeneity. Seven studies included in the “overall”, five in the <3 μL/dL, and two in the >3 μL/dL meta-analysis. | He et al. 2019[20] |
| Manganese | Postnatal | There was an association between manganese exposure and ADHD. | 3 | <1k | Moderate heterogeneity. | Nilsen and Tulve 2020[19] |
| Manganese | Childhood | Manganese levels were higher in ADHD children when studies of blood levels and hair levels were combined. However, when only studies investigating blood levels were included, there was no difference. | 4 | >1k | Moderate heterogeneity. Four studies included in the combined and three in the blood meta-analysis. | Shih et al. 2018[21] |
| Mercury | Postnatal | There was an association between exposure to mercury and ADHD. | 3 | >2k | Small heterogeneity. | Nilsen and Tulve 2020[19] |
| Mercury | Prenatal and Postnatal | Significant association was observed for environmental methylmercury exposure and risk of ADHD. Prenatal and early infancy thimerosal exposure did not. | 6 | >250k | Small to moderate heterogeneity. Only two studies with three datasets in the methylmercury and four studies with seven datasets in the thimerosal exposure meta-analysis. | Yoshimasu et al. 2014[22] |
| Nitrogen oxides | Prenatal and Postnatal | Not significant. | 9 | >100k | Moderate heterogeneity. Five studies with six datasets. | Zhang et al. 2020[23] |
| Organic contaminants | Postnatal | Not significant (organophosphate pesticides, polychlorinated biphenyls, pyrethroid pesticides, and trichlorophenol). | 6 | >5k | Small heterogeneity. | Nilsen and Tulve 2020[19] |
| Particulate matter | Prenatal and Postnatal | Not significant. | 6 | >100k | Moderate heterogeneity. Six studies with eight datasets. | Zhang et al. 2020[23] |
| Perfluoroalkyl Substances | Prenatal and Postnatal | Not significant in any time point evaluated | 9 | >4k | Small heterogeneity. Results from stratified analyses suggest potential differential effects of perfluoroalkyl substances related to child sex and maternal education. | Forns et al. 2020[24] |
| Phthalate/plasticizer exposures | Postnatal | The exposure to phthalate and plasticizers was associated with ADHD. | 5 | >2k | High heterogeneity. The amount of heterogeneity in the analysis is likely due to the ten different compounds compared. | Nilsen and Tulve 2020[19] |
| Polycyclic aromatic hydrocarbons | In utero Childhood | Not significant in the main analysis. The association was significant in the sensitivity analysis after the exclusion of one study. | 5 | >2k | High heterogeneity. Small heterogeneity when results of four studies from the same research group were meta-analyzed. | Kalantary et al. 2020[25] |
| Polycyclic aromatic hydrocarbons | Prenatal and Postnatal | Not significant. | 3 | >1k | Small heterogeneity. Three studies with eight datasets. | Zhang et al. 2020[23] |
| Smoking | In utero | Maternal smoking during pregnancy was related to an increased risk of ADHD. Only prospective cohorts were included. | 12 | >1M | High heterogeneity. Adjusting for maternal age increased the association. Studies published after 2010 presented a stronger association. | He et al. 2020[26] |
| Smoking | Prenatal | Either prenatal exposure to maternal smoking or smoking cessation during 1st trimester was associated with ADHD. | 27 | >3M | Small to high heterogeneity. Associations remained after adjusting for parental psychiatric history and social socioeconomic status. Number of studies included in each meta-analysis varied. | Dong et al. 2018[27] |
| Smoking | Prenatal | Maternal smoking during pregnancy was associated with increased risk of ADHD. The risk was greater for children whose mothers were heavy smokers. | 20 | >3M | High heterogeneity. Six studies included in the meta-analysis of heavy smokers. Studies with a sibling design (three studies) did not present a significant result in the subgroup analysis. | Huang et al. 2018[28] |
| Smoking (Secondhand) | Prenatal | Two separate meta-analyses showed evidence of paternal smoking during pregnancy being associated with ADHD. | 5 | >180k | Small to moderate heterogeneity. Three studies included in the OR and two in the RR meta-analysis. | Easey and Sharp 2021[29] |
| Smoking (Secondhand) | Postnatal | Secondhand smoking increased the risk of ADHD. | 9 | >100k | Moderate heterogeneity. | Huang et al. 2021[30] |
| Smoking (Secondhand) | Postnatal | The exposure to cigarette smoke (evaluated to cotinine levels and parental surveys) was associated with ADHD. | 6 | >9k | Small heterogeneity. | Nilsen and Tulve 2020[19] |

**Additional file 1: Table S2 Meta-analyses summarizing associations with biomarkers and nutritional factors**

| **Study factor** | **Matrix** | Age group at assessment | **Main finding(s)** | **N studies** | **Sample size** | **Observations** | **Ref.** |
| --- | --- | --- | --- | --- | --- | --- | --- |
| Adrenergic pathway | Urine and  Plasma | Children | Not significant for urinary and plasma epinephrine (EPI) levels. Urinary metanephrine (M) levels were elevated in ADHD. | 12 | <1k | Small to high heterogeneity. Six studies included in the urinary EPI, four in the plasma EPI, and five in the M meta-analysis. | Scassellati et al. 2012[31] |
| Brain-derived neurotrophic factor (BDNF) | Serum and Plasma | Children and Adolescents | Peripheral BDNF levels did not differ. However, BDNF levels were higher in males with ADHD. | 10 | >1k | Moderate to high heterogeneity. Three studies included in the male and three in the female meta-analysis. | Zhang et al. 2018[32] |
| Cortisol | Saliva and Blood | Children and Adolescents | Patients with ADHD presented lower “any time-point during the day” “cumulative levels” of cortisol throughout the day. Moreover, morning cortisol levels were lower in ADHD, while there was no difference for the afternoon cortisol levels. | 19 | >1k | Moderate to high heterogeneity. Nineteen studies included in the “any time-points”, six in the cumulative levels, fourteen in the morning levels, and nine in the afternoon levels meta-analysis. | Chang et al. 2021[33] |
| Cortisol | Saliva | Adults | Not significant. | 3 | <1k | High heterogeneity. | Bonvicini et al. 2016[34] |
| Cortisol | Saliva | Children | Patients with ADHD presented lower cortisol levels. | 9 | <1k | Small heterogeneity. One study was excluded. | Scassellati et al. 2012[31] |
| Cortisol reactivity | Saliva | Children, Adolescents, and Adults | Not significant. | 12 | >1k | High heterogeneity. | Kamradt et al. 2018[35] |
| Dietary patterns | NA | Children and Adolescents | The “healthy” dietary pattern decreased the risk of ADHD, whereas the “Western” and “junk food” dietary patterns increased it. | 6 | >8k | Small to high heterogeneity. Five studies included in the “healthy”, five in the “junk food”, and two in the “Western” meta-analysis. | Shareghfarid et al. 2020[36] |
| Dietary patterns | NA | Children and Adolescents | The “healthy” dietary pattern decreased the risk of ADHD, whereas the “unhealthy” dietary pattern increased it. | 14 | >30k | Moderate to high heterogeneity. Fourteen studies included in the “unhealthy” and eleven in the “healthy” meta-analysis. | Del-Ponte et al. 2019[37] |
| Dopaminergic pathway | Urine | Children | Not significant for dopamine (DA) and homovanillic acid (HVA) levels. | 11 | <1k | Small to moderate heterogeneity. Four studies included in the DA and nine in the HVA levels meta-analysis. | Scassellati et al. 2012[31] |
| Folic acid | Serum and Plasma | Children and Adolescents | Folic acid levels were higher in ADHD. | 5 | >1k | High heterogeneity. | Prades et al. 2022[38] |
| Inflammatory markers | Blood | Children and Adolescents | Patients with ADHD presented lower tumor necrosis factor-alpha (TNF-α). There were no differences for interleukins (IL-1β, IL-6, and IL-10). | 4 | <1k | Small (TNF- α) to high heterogeneity. Three studies included in the TNF-α, three in the IL-1β, four in the IL-6, and three in the IL-10 levels meta-analysis | Chang et al. 2021[33] |
| Iron related | Serum | Children and Adolescents | Serum ferritin levels were lower in ADHD. Iron deficiency was associated with ADHD. However, there were no correlations with serum iron or transferrin levels. | 17 | >5k | Moderate to high heterogeneity. Thirteen studies included in the ferritin, six in the iron, two in the transferrin levels, and three/four in the iron deficiency meta-analysis. | Tseng et al. 2018[39] |
| Iron related | Serum | Children and Adolescents | Serum ferritin levels were lower in ADHD cases. However, there was no correlation between serum iron levels and ADHD. | 11 | >1k | High heterogeneity. Ten studies included in the ferritin and six in the iron levels meta-analysis. | Wang et al. 2017[40] |
| Iron related | Serum | Children | Serum ferritin levels were lower in ADHD cases. | 7 | >1K | High heterogeneity. One study was excluded. | Scassellati et al. 2012[31] |
| Iron related | Serum | Children | Serum ferritin levels were lower in ADHD cases. | 5 | <1k | High heterogeneity. | Tan et al. 2011[41] |
| Magnesium | Serum | Children, Adolescents, and Adults | Individuals with ADHD presented lower serum magnesium. | 7 | >3k | High heterogeneity. | Effatpanah et al. 2019[42] |
| Magnesium | Serum, Plasma, and Hair | Children and Adolescents | Individuals with ADHD presented lower magnesium in whole blood, serum, and hair. | 12 | >5k | High heterogeneity. Eight studies included in the whole blood, six in the serum, and four in the hair meta-analysis. | Huang et al. 2019[43] |
| Noradrenergic pathway | Urine, Plasma, and Platelet | Children | Patients with ADHD presented higher urinary levels of norepinephrine (NE) and normetanephrine (NM). Lower urinary 3-methoxy-4-hydroxyphenylethylene glycol (MHPG) and platelet monoamine-oxidase (MAO) levels were also detected. There was no difference detected for plasma NE levels. | 26 | <1k | Small to high heterogeneity. Seven studies included in the urinary NE, four in the plasma NE, six in the NM, fifteen in the MHPG, and six in the MAO meta-analysis. | Scassellati et al. 2012[31] |
| Polyunsaturated fatty acids (PUFAs) | Irrespective of tissue source | Children and Adolescents | Children and adolescents with ADHD presented lower levels of docosahexaenoic acid (DHA), eicosapentaenoic acid (EPA), arachidonic acid (AA) and total Omega-3 PUFAs, but not Omega-6 PUFAs. | 9 | <1k | Small to high heterogeneity. Eight studies included in the DHA, eight in the EPA, eight in the AA, and seven in the n-3 PUFAs levels meta-analysis. The results presented for EPA, AA, and n-3 PUFAs were significant only after the exclusion of one study. | Chang et al. 2018[44] |
| Polyunsaturated fatty acids (PUFAs) | Red blood cell and Plasma | Adults | Adults with ADHD presented lower levels of docosahexaenoic acid (DHA). The other PUFAs were not significant (arachidonic acid, dihomogammalinolenic acid, and eicosapentaenoic acid). | 3 | <1k | Small (DHA meta-analysis) to high heterogeneity. | Bonvicini et al. 2016[34] |
| Polyunsaturated fatty acids (PUFAs) | Red blood cell and Plasma | Children and Adolescents | Patients with ADHD presented higher omega-6/omega-3 and arachidonic acid (AA)/eicosapentaenoic acid (EPA) ratios | 5 | <1k | Small to high heterogeneity. Five studies included in the Omgea-6/Omega-3 and three in the AA/EPA meta-analysis. | Lachance et al. 2016[45] |
| Polyunsaturated fatty acids (PUFAs) | Red blood cell and Plasma | Children, Adolescents and Adults | Individuals with ADHD presented lower levels of Omega-3 PUFAs (combined) as well as docosahexaenoic acid (DHA) and eicosapentaenoic acid (EPA) levels alone. | 9 | <1k | Small heterogeneity. | Hawkey and Nigg 2014[46] |
| Serotonergic Pathway | Urine | Children | Not significant for the 5-hdryxitriptamine metabolite 5-hydroxyindoleacetic acid | 4 | <1k | Moderate heterogeneity. | Scassellati et al. 2012[31] |
| Sugars | NA | Children and Adolescents | There was an association between sugars consumption and ADHD. | 7 | >25k | High heterogeneity. The association was potentially driven by sugar-sweetened beverages consumption. | Farsad-Naeimi et al. 2020[47] |
| Vitamin B12 | Serum and Plasma | Children and Adolescents | Vitamin B12 levels were decreased in ADHD. | 5 | >1k | High heterogeneity. | Prades et al. 2022[38] |
| Vitamin D | Serum | Children and Adolescents | Lower vitamin D levels in patients with ADHD. | 8 | >11k | High heterogeneity. | Kotsi et al. 2019[48] |
| Vitamin D | Cord blood and Maternal blood | Children, Adolescents and Adults | There was an inverse relationship between ADHD and vitamin D levels in the maternal blood in pregnancy or newborn blood at birth. | 5 | >4k | Small heterogeneity. | García-Serna and Morales 2019[49] |
| Vitamin D | Serum, Cord blood, and Maternal blood | Children, Adolescents and Adults | Patients with ADHD presented lower serum levels of vitamin D. Lower vitamin D status was associated with the likelihood of ADHD. Perinatal suboptimal vitamin D levels were associated with a higher risk of ADHD in later life. | 13 | >10k | Small to high heterogeneity. Nine studies included in the serum, five in the vitamin D status, and four in the perinatal meta-analysis. It should be noted that the association found in perinatal studies was sensitive to one of the included investigations. | Khoshbakht et al. 2018[50] |
| Zinc | Serum, Plasma, Urine, and Hair | Children | Lower serum zinc levels in patients with ADHD. | 6 | >1k | High heterogeneity. One study was excluded. | Scassellati et al. 2012[31] |
| Zinc | Serum, Plasma, and Hair | Children, Adolescents, and Adults | Not significant. | 22 | >2k | High heterogeneity. Fourteen studies included in the serum/plasma and eight in the hair meta-analysis. Sensitivity analysis showed that exclusion of one study from the analysis changed the overall effect size towards to lower serum zinc levels in patients with ADHD. | Ghoreishy et al. 2021[51] |
| Zinc | Serum, Plasma, and Hair | Children and Adolescents | Not significant. | 11 | >1k | High heterogeneity. Eight studies included in the serum/plasma and three in the hair meta-analysis. Sensitivity analysis showed that exclusion of one study from the analysis changed the overall effect size towards to lower serum zinc levels in patients with ADHD. | Luo et al. 2020[52] |
| Zinc | Serum | Children and Adolescents | Lower serum zinc levels in patients with ADHD. | 17 | >5k | High heterogeneity. | Sum et al. 2015[53] |

**Additional file 1: Table S3 Meta-analyses summarizing associations with parental, newborn, and lifespan-related factors**

| Study factor | Age group at assessment | Main finding(s) | N studies | Sample size | Observations | Ref. |
| --- | --- | --- | --- | --- | --- | --- |
| Allergic conjunctivitis | Children, Adolescents, and Adults | Allergic conjunctivitis in childhood was associated with ADHD. | 3 | >40k | High heterogeneity. | Miyazaki et al. 2017[54] |
| Allergic rhinitis | Children and Adolescents | Allergic rhinitis in childhood was associated with ADHD. | 3 | >40k | High heterogeneity. | van der Schans et al. 2017[55] |
| Allergic rhinitis | Children, Adolescents, and Adults | Allergic rhinitis in childhood was associated with ADHD. | 5 | >50k | High heterogeneity. | Miyazaki et al. 2017[54] |
| Asthma | Children and Adolescents | Asthma in childhood was associated with ADHD. | 23 | >400k | Moderate heterogeneity. | Kaas et al. 2021[56] |
| Asthma | Children, Adolescents, and Adults | Asthma in childhood was associated with ADHD. The association was also detected in a large populational independent study. | 84/49^a^ | >4M | Moderate to high heterogeneity. Twenty-eight cross-sectional studies included in the unadjusted and twelve in the adjusted meta-analysis. | Cortese et al. 2018[57] |
| Asthma | Children and Adolescents | Asthma in childhood was associated with ADHD. | 6 | >60k | Small heterogeneity. | van der Schans et al. 2017[55] |
| Asthma | Children, Adolescents, and Adults | Asthma in childhood was associated with ADHD. | 5 | >50k | Moderate heterogeneity | Miyazaki et al. 2017[54] |
| Atopic dermatitis (eczema) | Children and Adolescents | Atopic dermatitis in childhood was associated with ADHD. | 19 | >1M | High heterogeneity. | Xie et al. 2019[58] |
| Atopic dermatitis (eczema) | Children and Adolescents | Atopic dermatitis in childhood was associated with ADHD. | 6 | >50k | Small heterogeneity. | van der Schans et al. 2017[55] |
| Atopic dermatitis (eczema) | Children, Adolescents, and Adults | Atopic dermatitis in childhood was associated with ADHD. | 5 | >50k | High heterogeneity. | Miyazaki et al. 2017[54] |
| Caesarean delivery | Children and adolescents | Caesarean delivery was associated with increase in the risk of ADHD. The association remained for both infants born after elective and emergency caesareans. | 9 | >2M | Small to moderate heterogeneity. Nine studies were included in the “all estimates”, six in the elective caesarean, and six in the emergency caesarean meta-analysis. The association was only marginally significant when data from siblings from other pregnancies were pooled (two studies/four estimates), implying that the association was potentially due to confounding. | Xu et al. 2020[59] |
| Caesarean delivery | Children, Adolescents and Adults | Caesarean delivery was associated with increase in the risk of ADHD. The association remained for both infants born after elective and emergency caesareans. | 13 | >3M | Small to high heterogeneity. Thirteen studies included in the “all estimates”, four in the elective caesarean, and four in the emergency caesarean meta-analysis. | Zhang et al. 2019[60] |
| Caesarean delivery | Children and Adolescents | Not associated in the adjusted and associated with ADHD in the unadjusted meta-analysis. | 4 | >60k | Small heterogeneity. Two studies included in the adjusted and three in the unadjusted meta-analysis. | Curran et al. 2015[61] |
| Child younger than school classmates | Children and Adolescents | Children who were relatively young when started school (owing to month of birth) had an increased risk of ADHD. | 11 | >500k | High heterogeneity. | Hsu et al. 2021[62] |
| Child younger than school classmates | Children and Adolescents | Children and adolescents who are relatively younger compared with their classmates presented a higher risk of receiving an ADHD diagnosis. | 25/30^b^ | >8M | High heterogeneity. A set of sensitivity analyses showed that results were consistent even changing several methodological choices. No difference by sex was found. | Caye et al. 2020[63] |
| Childhood eating disorder (ED) | Children and adolescents | ED was associated with ADHD. | 6 | >20k | Small to high heterogeneity. Five studies included in the ADHD and two in the ED clinical background meta-analysis. | Nazar et al. 2016[64] |
| Childhood obesity | Children and Adolescents | Childhood obesity was associated with ADHD. | 30 | >600k | High heterogeneity. | Cortese et al. 2016[65] |
| Food allergy | Children and Adolescents | Not associated | 3 | >8k | Small heterogeneity. | Miyazaki et al. 2017[54] |
| Hypertensive disorders of pregnancy | Children, Adolescents, and Adults | Maternal hypertensive disorders (mainly preeclampsia) increased the risk of ADHD | 9 | >1M | Small to moderate heterogeneity. Adjusted estimates remained relatively unchanged (six studies). | Maher et al. 2018[66] |
| Hypertensive disorders of pregnancy | Children, Adolescents, and Adults | Preeclampsia increased the risk of ADHD. | 8 | >1M | Small heterogeneity. | Zhu et al. 2016[67] |
| Maternal breastfeeding (BF) | Children | The mean BF duration was shorter in children with ADHD. Furthermore, the ADHD children stopped BF earlier than controls (higher rate of less than 3 months BF in ADHD and lower rate of between 6-12 months BF or more than 12 months BF in ADHD). | 11 | >90k | Moderate to high heterogeneity. Four studies included in the BF duration, seven “less than 3 months”, three in the “6-12 months”, and three in the “>12 months” meta-analysis. | Tseng et al. 2019[68] |
| Maternal breastfeeding (BF) | Children and Adolescents | Individuals with any maternal BF had a lower incidence of ADHD. Children breastfed for over 1 month, over 3 months, over 6 months, and over 12 months had a lower incidence of ADHD. | 12 | >100k | Small to moderate heterogeneity. Seven studies included in the “any BF”, three in the “over 1 month”, five in the “over three months”, six in the “over six months”, and four in the “over twelve months” meta-analysis. | Zeng et al. 2018[69] |
| Maternal cell phone use during pregnancy | Children | Children from non-users were at a lower risk of hyperactivity and inattention problems. Mothers who were medium or high cell phone users during pregnancy were at higher risk to have children with hyperactivity and inattention problems. | 5 | >80k | Small heterogeneity. | Birks et al. 2017[70] |
| Maternal diabetes mellitus (DM) | Children | Not associated. | 8 | >30k | Small heterogeneity. Five out eight studies were meta-analyzed. Three studies were excluded because the authors were unable to verify that pregestational DM had been excluded from the controls. | Rowland and Wilson 2021[71] |
| Maternal diabetes mellitus (DM) | Children, Adolescents, and Adults | Maternal diabetes mellitus was associated with an increased risk of ADHD. The association was evident for pregestational DM, including preexisting type 1 DM, but not for gestational DM. | 9 | >5M | Small to high heterogeneity. Nine studies included in the adjusted maternal DM, five in the adjusted pregestational DM, four in the gestational DM and four in the adjusted type 1 DM meta-analysis. | Zeng et al. 2020[72] |
| Maternal diabetes mellitus (DM) | Children and Adolescents | Maternal pregestational DM was associated with ADHD. | 7 | >3M | Small to high heterogeneity. Not significant when only case-control studies (two) or studies evaluating gestational DM (five) were combined. | Guo et al. 2020[73] |
| Maternal diabetes mellitus (DM) | Children, Adolescents, and Adults | Maternal DM increased the risk of ADHD after combining cohort studies. Effect sizes were higher for gestational DM. | 9 | >4M | Small to high heterogeneity. Not significant after combining case control studies Five studies included in the case-control studies, six in the cohort studies, and four in the gestational DM meta-analysis. | Zhao et al. 2019[74] |
| Maternal diabetes mellitus (DM) | Children, Adolescents, and Adults | Maternal pre-existing diabetes was associated with ADHD. | 2 | >1M | Small heterogeneity. | Yamamoto et al. 2019[75] |
| Maternal dietary quality | Children | Maternal low-quality and proinflammatory diet during pregnancy increased the risk of ADHD. | 4 | >10k | Small to moderate heterogeneity. | Polanska et al. 2021[76] |
| Maternal folic acid supplementation | Children | Maternal folic acid supplementation during pregnancy was associated with reduced risk for ADHD in the offspring. | 6 | >25k | Small heterogeneity. | Chen et al. 2021[77] |
| Maternal infection during pregnancy | Children and Adolescents | Maternal infection was associated with risk of ADHD. In subgroup analyses, the association remained for maternal genitourinary (GU) infection. | 8 | >500k | Small to high heterogeneity. Six studies included in the maternal infection and three in the GU meta-analysis. There was no difference in the risk of ADHD between siblings who were or were not exposed to infection in utero (two studies meta-analyzed), implying that the association was due to confounding. | Zhu et al. 2022[78] |
| Maternal moderate coffee consumption during pregnancy | Children | Not significant | 3 | >7k | High heterogeneity. | Li et al. 2019[79] |
| Maternal pre-pregnancy overweight/obesity | Children and Adolescents | Maternal overweight and obesity were both associated with an increased risk of ADHD. | 8 | >700k | Small heterogeneity. The association between maternal pre-pregnancy overweight/obesity and risk of ADHD in offspring was ascribed to unmeasured familial confounding in a quasi-experimental family-based study. | Li et al. 2020[80] |
| Maternal pre-pregnancy overweight/obesity | Children and Adolescents | Maternal overweight and obesity were both associated with an increased risk of ADHD. | 10 | >1M | Small to moderate heterogeneity. Two studies included in the HR overweight, seven in the OR overweight, three in the HR obesity, and eight in the OR obesity meta-analysis. | Jenabi et al. 2019[81] |
| Maternal pre-pregnancy overweight/obesity | Children and Adolescents | Maternal overweight and obesity were both associated with an increased risk of ADHD. | 8 | >10k | Moderate to high heterogeneity. Six studies included in the overweight, seven in the obese, and eight in the “overweight + obese combined” meta-analysis. | Sanchez et al. 2018[82] |
| Maternal stress during pregnancy | Children and Adolescents | Maternal stress during pregnancy was associated with ADHD. | 12 | >1M | High heterogeneity. Eleven studies included in the unadjusted and seven in the adjusted meta-analysis. | Manzari et al. 2019[83] |
| Maternal thyroid disfunction | Children | Both maternal hyperthyroidism and hypothyroidism were associated with ADHD. | 4 | >3M | Small heterogeneity. Two studies included in the hyperthyroidism and four in the hypothyroidism meta-analysis. | Ge et al. 2020[84] |
| Maternal thyroid disfunction | Children | Not associated. Inconsistent findings related to gestational age effects. | 3 | >7k | Small to moderate heterogeneity. There studies included in the FT4 and three in the TSH levels meta-analysis. | Levie et al. 2019[85] |
| Maternal thyroid disfunction | Children | Not associated. | 3 | >5k | High heterogeneity. Two studies included in the subclinical hypothyroidism and two in the hypothyroxinaemia meta-analysis. | Thompson et al. 2018[86] |
| Oxytocin use for labor induction | Children, Adolescents and Adults | Not associated | 7 | >2M | Moderate to high heterogeneity. Four studies included in the OR and three in the RR meta-analysis. | Jenabi et al. 2021[87] |
| Oxytocin use for labor induction | Children, Adolescents, and Adults | Not associated. | 3 | >500k | High heterogeneity. | Lonfeldt et al. 2019[88] |
| Paternal diabetes mellitus (DM) | Children and Adolescents | Paternal type 1 DM was associated with ADHD. | 3 | >4M | Small heterogeneity. | Zeng et al. 2020[72] |
| Perinatal hypoxic-ischemic conditions | Children, Adolescents, and Adults | Apgar score <7 at 5 minutes and breech/transverse presentations were associated with risk of ADHD. Prolapsed nuchal cord did not. | 10 | >9M | Small to moderate heterogeneity. Six in the Apgar <7 at 5 minutes, four in the breech/transverse presentations, and three in the prolapsed nuchal cord meta-analysis. | Zhu et al. 2016[67] |
| Preterm / Birth Weight | Adults | Not associated in the individual participant data meta-analysis (ADHD symptoms levels were similar across groups). Whereas in a register-linkage larger study (>700k), adults born preterm had a higher risk of ADHD. Among preterm individuals, as gestation length and standard deviation birth weight z-score increased, the risk of ADHD decreased | 8/1^c^ | >3k | Heterogeny was not assessed (it is an individual participant data meta-analysis). Eight studies included in the total ADHD symptoms, ADHD symptoms above versus below clinical cut-off, inattention, and hyperactivity meta-analysis. Six studies included in the attention problems meta-analysis. | Robinson et al. 2022[89] |
| Preterm / Birth Weight | Children, Adolescents, and Adults | Very Preterm / Very Low Birth Weight subjects presented higher risk of ADHD. The increase in meeting criteria for ADHD persisted into adulthood, albeit at lower rates than observed in childhood. | 7 | >1k | Heterogeny was not assessed (individual participant data meta-analysis). | Anderson et al. 2021[90] |
| Preterm / Birth Weight | Children, Adolescents, and Adults | Very Preterm / Very Low Birth Weight subjects presented a higher ADHD risk (the more extreme the cases, the higher the risk). There were also effects on inattention, hyperactivity and impulsivity, and combined ADHD symptoms. | 34 | >4k | Small to high heterogeneity. Twelve studies included in the categorical ADHD, nine in the inattention, thirteen in the hyperactivity/impulsivity, and twenty-four in the combined symptoms meta-analysis. | Franz et al. 2018[91] |
| Preterm / Birth Weight | Children, Adolescents, and Adults | Birth weight presented an association with ADHD symptoms such that individuals born at lower birth weights manifested greater symptoms. | 85/93^d^ | >4M | High heterogeneity. Ninety-three studies included in the “all studies”, eighty-five in the “unadjusted studies”, and eight in the “adjusted studies” meta-analysis. Several variables contributed significantly to heterogeneity in effect sizes. | Momany et al. 2018[92] |
| Preterm / Birth Weight | Children | ADHD was associated with preterm birth with a differential effect observed according to the severity of prematurity. | 10 | >45k | Moderate heterogeneity. | Allotey et al. 2018[93] |
| Regulatory problems (crying, sleeping, and feeding) | Children and Adolescents | Any regulatory, crying, and sleeping problems were associated with ADHD. | 22 | >1k | Small to high heterogeneity. Thirteen studies included in the any regulatory, six in the crying, and two in the sleeping problems meta-analysis. | Hemmi et al. 2011[94] |
| Retinal nerve fiber/ganglion cell layer (RNFL/GCL) thickness | Children/ Adolescents | A reduction in global RNFL thickness was associated with ADHD. The global GCL thickness was not associated with ADHD. | 4 | <1k | Small to high heterogeneity. Four studies included in the RNFL and two in the GCL meta-analysis. | Li et al. 2021[95] |
| Socioeconomic status (SES) | Children and Adolescents | Individuals of a mother or a father with low education level presented increased risk of ADHD. Individuals living with single parents or in families with low SES presented increased risk of ADHD. | 15 | >200k | Moderate to high heterogeneity. Six studies included in the mother education, three in the father education, ten in the single family, and four in the index of SES meta-analysis. | Russell et al. 2016[96] |
| Toxoplasma gondii infection | Children, Adolescents, and Adults | Not significant. | 7 | >6k | Small to moderate heterogeneity. Seven studies included in the IgG, and four in the IgM meta-analysis. There was publication bias. | Nayeri et al. 2020[97] |
| Traumatic brain injury (TBI) | Children, Adolescents, and Adults | TBI in the overall analysis was not associated with ADHD. However, there were associations between severe TBI and ADHD after T1 (time 1-year or less postinjury) and T2 (time more than 1-year postinjury). | 24 | >55k | Heterogeneity was not evaluated in the standard way. Nineteen studies included in the overall, seven in the T1 and seven in the T2 meta-analysis. | Asarnow et al. 2021[98] |
| Traumatic brain injury (TBI) | Children, Adolescents, and Adults | There was an association between ADHD and mild TBI, which was significant when limited to studies that reported on ADHD after TBI and when the direction of the association was not specified, but not for studies that reported TBI after ADHD. | 5/8^e^ | >10k | Small heterogeneity. Five datasets were included in the “unknown ADHD/TBI sequence”, two in the “ADHD prior to TBI”, and two in the “ADHD after TBI” meta-analysis. | Adeyemo et al. 2014[99] |

^a^Eighty-four articles and forty-nine datasets. ^b^Twenty-five studies with thirty samples. ^c^Eight studies and an additional register-linkage study. ^d^Eighty-five studies with ninety-three samples. ^e^Five studies and eight datasets.

**References**

1. Alemany S, Avella-García C, Liew Z, García-Esteban R, Inoue K, Cadman T, et al. Prenatal and postnatal exposure to acetaminophen in relation to autism spectrum and attention-deficit and hyperactivity symptoms in childhood: Meta-analysis in six European population-based cohorts. Eur J Epidemiol. 2021;36:993–1004.

2. Masarwa R, Platt RW, Filion KB. Acetaminophen use during pregnancy and the risk of attention deficit hyperactivity disorder: A causal association or bias? Paediatr Perinat Epidemiol. 2020;34:309–317.

3. Gou X, Wang Y, Tang Y, Qu Y, Tang J, Shi J, et al. Association of maternal prenatal acetaminophen use with the risk of attention deficit/hyperactivity disorder in offspring: A meta-analysis. Aust N Z J Psychiatry. 2019;53:195–206.

4. Masarwa R, Levine H, Gorelik E, Reif S, Perlman A, Matok I. Prenatal Exposure to Acetaminophen and Risk for Attention Deficit Hyperactivity Disorder and Autistic Spectrum Disorder: A Systematic Review, Meta-Analysis, and Meta-Regression Analysis of Cohort Studies. Am J Epidemiol. 2018;187:1817–1827.

5. San Martin Porter M, Maravilla JC, Betts KS, Alati R. Low-moderate prenatal alcohol exposure and offspring attention-deficit hyperactivity disorder (ADHD): systematic review and meta-analysis. Arch Gynecol Obstet. 2019;300:269–277.

6. Wetherill L, Foroud T, Goodlett C. Meta-Analyses of Externalizing Disorders: Genetics or Prenatal Alcohol Exposure? Alcohol Clin Exp Res. 2018;42:162.

7. Khoury JE, Jamieson B, Milligan K. Risk for Childhood Internalizing and Externalizing Behavior Problems in the Context of Prenatal Alcohol Exposure: A Meta-Analysis and Comprehensive Examination of Moderators. Alcohol Clin Exp Res. 2018;42:1358–1377.

8. Duong QA, Pittet LF, Curtis N, Zimmermann P. Antibiotic exposure and adverse long-term health outcomes in children: a systematic review and meta-analysis. J Infect. 2022. 2022. https://doi.org/10.1016/J.JINF.2022.01.005.

9. Ai Y, Zhao J, Shi J, Zhu TT. Antibiotic exposure and childhood attention-deficit/hyperactivity disorder: systematic review and meta-analysis. Psychopharmacology (Berl). 2021;238:3055–3062.

10. Yu H ying, Zhou Y yue, Pan L ya, Zhang X, Jiang H yin. Early Life Antibiotic Exposure and the Subsequent Risk of Autism Spectrum Disorder and Attention Deficit Hyperactivity Disorder: A Systematic Review and Meta-Analysis. J Autism Dev Disord. 2021:1–11.

11. Halvorsen A, Hesel B, Østergaard SD, Danielsen AA. In utero exposure to selective serotonin reuptake inhibitors and development of mental disorders: a systematic review and meta-analysis. Acta Psychiatr Scand. 2019;139:493–507.

12. Leshem R, Bar-Oz B, Diav-Citrin O, Gbaly S, Soliman J, Renoux C, et al. Selective Serotonin Reuptake Inhibitors (SSRIs) and Serotonin Norepinephrine Reuptake Inhibitors (SNRIs) During Pregnancy and the Risk for Autism spectrum disorder (ASD) and Attention deficit hyperactivity disorder (ADHD) in the Offspring: A True Effect o. Curr Neuropharmacol. 2021;19:896–906.

13. Jiang HY, Peng CT, Zhang X, Ruan B. Antidepressant use during pregnancy and the risk of attention-deficit/hyperactivity disorder in the children: a meta-analysis of cohort studies. BJOG An Int J Obstet Gynaecol. 2018;125:1077–1084.

14. Morales DR, Slattery J, Evans S, Kurz X. Antidepressant use during pregnancy and risk of autism spectrum disorder and attention deficit hyperactivity disorder: Systematic review of observational studies and methodological considerations. BMC Med. 2018;16:1–14.

15. Man KKC, Chan EW, Ip P, Coghill D, Simonoff E, Chan PKL, et al. Prenatal antidepressant exposure and the risk of attention-deficit hyperactivity disorder in children: A systematic review and meta-analysis. Neurosci Biobehav Rev. 2018;86:1–11.

16. Rochester JR, Bolden AL, Kwiatkowski CF. Prenatal exposure to bisphenol A and hyperactivity in children: a systematic review and meta-analysis. Environ Int. 2018;114:343–356.

17. Song J, Li H, Wang Y, Niu C. Does Exposure to General Anesthesia Increase Risk of ADHD for Children Before Age of Three? Front Psychiatry. 2021;12:1659.

18. Sun J jun, Zhu C yan, Jiang H yin. Exposure to general anaesthesia in childhood and the subsequent risk of attention-deficit hyperactivity disorder: A meta-analysis of cohort studies. Asian J Psychiatr. 2021;62:102708.

19. Nilsen FM, Tulve NS. A systematic review and meta-analysis examining the interrelationships between chemical and non-chemical stressors and inherent characteristics in children with ADHD. Environ Res. 2020;180:108884.

20. He J, Ning H, Huang R. Low blood lead levels and attention-deficit hyperactivity disorder in children: a systematic review and meta-analysis. Environ Sci Pollut Res. 2019;26:17875–17884.

21. Shih JH, Zeng BY, Lin PY, Chen TY, Chen YW, Wu CK, et al. Association between peripheral manganese levels and attention-deficit/hyperactivity disorder: a preliminary meta-analysis. Neuropsychiatr Dis Treat. 2018;14:1831–1842.

22. Yoshimasu K, Kiyohara C, Takemura S, Nakai K. A meta-analysis of the evidence on the impact of prenatal and early infancy exposures to mercury on autism and attention deficit/hyperactivity disorder in the childhood. Neurotoxicology. 2014;44:121–131.

23. Zhang M, Wang C, Zhang X, Song H, Li Y. Association between exposure to air pollutants and attention-deficit hyperactivity disorder (ADHD) in children: a systematic review and meta-analysis. Int J Environ Health Res. 2020;32:207–219.

24. Forns J, Verner MA, Iszatt N, Nowack N, Bach CC, Vrijheid M, et al. Early Life Exposure to Perfluoroalkyl Substances (PFAS) and ADHD: A Meta-Analysis of Nine European Population-Based Studies. Environ Health Perspect. 2020;128:20.

25. Kalantary RR, Jaffarzadeh N, Rezapour M, Hesami Arani M. Association between exposure to polycyclic aromatic hydrocarbons and attention deficit hyperactivity disorder in children: a systematic review and meta-analysis. Environ Sci Pollut Res. 2020;27:11531–11540.

26. He Y, Chen J, Zhu LH, Hua LL, Ke FF. Maternal Smoking During Pregnancy and ADHD: Results From a Systematic Review and Meta-Analysis of Prospective Cohort Studies. J Atten Disord. 2020;24:1637–1647.

27. Dong T, Hu W, Zhou X, Lin H, Lan L, Hang B, et al. Prenatal exposure to maternal smoking during pregnancy and attention-deficit/hyperactivity disorder in offspring: A meta-analysis. Reprod Toxicol. 2018;76:63–70.

28. Huang L, Wang Y, Zhang L, Zheng Z, Zhu T, Qu Y, et al. Maternal Smoking and Attention-Deficit/Hyperactivity Disorder in Offspring: A Meta-analysis. Pediatrics. 2018;141:e20172465.

29. Easey KE, Sharp GC. The impact of paternal alcohol, tobacco, caffeine use and physical activity on offspring mental health: a systematic review and meta-analysis. Reprod Health. 2021;18:1–11.

30. Huang A, Wu K, Cai Z, Lin Y, Zhang X, Huang Y. Association between postnatal second-hand smoke exposure and ADHD in children: a systematic review and meta-analysis. Environ Sci Pollut Res Int. 2021;28:1370–1380.

31. Scassellati C, Bonvicini C, Faraone S V., Gennarelli M. Biomarkers and Attention-Deficit/Hyperactivity Disorder: A Systematic Review and Meta-Analyses. J Am Acad Child Adolesc Psychiatry. 2012;51:1003-1019.e20.

32. Zhang J, Luo W, Li Q, Xu R, Wang Q, Huang Q. Peripheral brain-derived neurotrophic factor in attention-deficit/hyperactivity disorder: A comprehensive systematic review and meta-analysis. J Affect Disord. 2018;227:298–304.

33. Chang JPC, Su KP, Mondelli V, Pariante CM. Cortisol and inflammatory biomarker levels in youths with attention deficit hyperactivity disorder (ADHD): evidence from a systematic review with meta-analysis. Transl Psychiatry 2021 111. 2021;11:1–10.

34. Bonvicini C, Faraone S V, Scassellati C. Attention-deficit hyperactivity disorder in adults: A systematic review and meta-analysis of genetic, pharmacogenetic and biochemical studies. Mol Psychiatry. 2016;21:872–884.

35. Kamradt JM, Momany AM, Nikolas MA. A meta-analytic review of the association between cortisol reactivity in response to a stressor and attention-deficit hyperactivity disorder. ADHD Atten Deficit Hyperact Disord. 2018;10:99–111.

36. Shareghfarid E, Sangsefidi ZS, Salehi-Abargouei A, Hosseinzadeh M. Empirically derived dietary patterns and food groups intake in relation with Attention Deficit/Hyperactivity Disorder (ADHD): A systematic review and meta-analysis. Clin Nutr ESPEN. 2020;36:28–35.

37. Del-Ponte B, Quinte GC, Cruz S, Grellert M, Santos IS. Dietary patterns and attention deficit/hyperactivity disorder (ADHD): A systematic review and meta-analysis. J Affect Disord. 2019;252:160–173.

38. Prades N, Varela E, Flamarique I, Deulofeu R, Baeza I. Water-soluble vitamin insufficiency, deficiency and supplementation in children and adolescents with a psychiatric disorder: a systematic review and meta-analysis. Https://DoiOrg/101080/1028415X20212020402. 2022:1–23.

39. Tseng P-T, Cheng Y-S, Yen C-F, Chen Y-W, Stubbs B, Whiteley P, et al. Peripheral iron levels in children with attention-deficit hyperactivity disorder: a systematic review and meta-analysis. Sci Rep. 2018;8:788.

40. Wang Y, Huang L, Zhang L, Qu Y, Mu D. Iron Status in Attention-Deficit/Hyperactivity Disorder: A Systematic Review and Meta-Analysis. PLoS One. 2017;12:e0169145.

41. Tan L-N, Wei H-Y, Yao-Dong Zhang, Ai-Lian Lu YL. Relationship between serum ferritin levels and susceptibility to attention deficit hyperactivity disorder in children: a Meta analysis. Zhongguo Dang Dai Er Ke Za Zhi. 2011. https://pubmed.ncbi.nlm.nih.gov/21924020/. Accessed 31 January 2022.

42. Effatpanah M, Rezaei M, Effatpanah H, Effatpanah Z, Varkaneh HK, Mousavi SM, et al. Magnesium status and attention deficit hyperactivity disorder (ADHD): A meta-analysis. Psychiatry Res. 2019;274:228–234.

43. Huang Y-H, Zeng B-Y, Li D-J, Cheng Y-S, Chen T-Y, Liang H-Y, et al. Significantly lower serum and hair magnesium levels in children with attention deficit hyperactivity disorder than controls: A systematic review and meta-analysis. Prog Neuropsychopharmacol Biol Psychiatry. 2019;90:134–141.

44. Chang JPC, Su KP, Mondelli V, Pariante CM. Omega-3 Polyunsaturated Fatty Acids in Youths with Attention Deficit Hyperactivity Disorder: a Systematic Review and Meta-Analysis of Clinical Trials and Biological Studies. Neuropsychopharmacology. 2018;43:534–545.

45. LaChance L, McKenzie K, Taylor VH, Vigod SN. Omega-6 to Omega-3 Fatty Acid Ratio in Patients with ADHD: A Meta-Analysis. J Can Acad Child Adolesc Psychiatry. 2016;25:87–96.

46. Hawkey E, Nigg JT. Omega-3 fatty acid and ADHD: blood level analysis and meta-analytic extension of supplementation trials. Clin Psychol Rev. 2014;34:496–505.

47. Farsad-Naeimi A, Asjodi F, Omidian M, Askari M, Nouri M, Pizarro AB, et al. Sugar consumption, sugar sweetened beverages and Attention Deficit Hyperactivity Disorder: A systematic review and meta-analysis. Complement Ther Med. 2020;53:102512.

48. Kotsi E, Kotsi E, Perrea DN. Vitamin D levels in children and adolescents with attention-deficit hyperactivity disorder (ADHD): a meta-analysis. Atten Defic Hyperact Disord. 2019;11:221–232.

49. García-Serna AM, Morales E. Neurodevelopmental effects of prenatal vitamin D in humans: systematic review and meta-analysis. Mol Psychiatry 2019 2510. 2019;25:2468–2481.

50. Khoshbakht Y, Bidaki R, Salehi-Abargouei A. Vitamin D Status and Attention Deficit Hyperactivity Disorder: A Systematic Review and Meta-Analysis of Observational Studies. Adv Nutr. 2018;9:9–20.

51. Ghoreishy SM, Ebrahimi Mousavi S, Asoudeh F, Mohammadi H. Zinc status in attention-deficit/hyperactivity disorder: a systematic review and meta-analysis of observational studies. Sci Reports 2021 111. 2021;11:1–10.

52. Luo J, Mo Y, Liu M. Blood and hair zinc levels in children with attention deficit hyperactivity disorder: A meta-analysis. Asian J Psychiatr. 2020;47.

53. Sum G-X, Huang B-H, Zhang Y-F. Relationship between serum zinc levels and attention deficit hyperactivity disorder in children. Zhongguo Dang Dai Er Ke Za Zhi. 2015. https://pubmed.ncbi.nlm.nih.gov/26412183/. Accessed 31 January 2022.

54. Miyazaki C, Koyama M, Ota E, Swa T, Mlunde LB, Amiya RM, et al. Allergic diseases in children with attention deficit hyperactivity disorder: A systematic review and meta-analysis. BMC Psychiatry. 2017;17:1–12.

55. van der Schans J, Çiçek R, de Vries TW, Hak E, Hoekstra PJ. Association of atopic diseases and attention-deficit/hyperactivity disorder: A systematic review and meta-analyses. Neurosci Biobehav Rev. 2017;74:139–148.

56. Kaas TH, Vinding RK, Stokholm J, Bønnelykke K, Bisgaard H, Chawes BL. Association between childhood asthma and attention deficit hyperactivity or autism spectrum disorders: A systematic review with meta-analysis. Clin Exp Allergy. 2021;51:228–252.

57. Cortese S, Sun S, Zhang J, Sharma E, Chang Z, Kuja-Halkola R, et al. Association between attention deficit hyperactivity disorder and asthma: a systematic review and meta-analysis and a Swedish population-based study. The Lancet Psychiatry. 2018;5:717–726.

58. Xie QW, Xiaolu D, Tang X, Chan CHY, Chan CLW. Risk of mental disorders in children and adolescents with atopic dermatitis: A systematic review and meta analysis. Front Psychol. 2019;10:1773.

59. Xu L lian, Zhang X, Zhou G lin, Jiang C min, Jiang H yin, Zhou Y yue. Meta-analysis found that studies may have overestimated Caesarean section risks for attention-deficit hyperactivity disorder by ignoring confounding factors. Acta Paediatr. 2020;109:258–265.

60. Zhang T, Sidorchuk A, Sevilla-Cermeño L, Vilaplana-Pérez A, Chang Z, Larsson H, et al. Association of Cesarean Delivery With Risk of Neurodevelopmental and Psychiatric Disorders in the Offspring: A Systematic Review and Meta-analysis. JAMA Netw Open. 2019;2:e1910236–e1910236.

61. Curran EA, O’Neill SM, Cryan JF, Kenny LC, Dinan TG, Khashan AS, et al. Research Review: Birth by caesarean section and development of autism spectrum disorder and attention-deficit/hyperactivity disorder: a systematic review and meta-analysis. J Child Psychol Psychiatry. 2015;56:500–508.

62. Hsu CW, Tseng PT, Tu YK, Lin PY, Hung CF, Liang CS, et al. Month of birth and mental disorders: A population-based study and validation using global meta-analysis. Acta Psychiatr Scand. 2021;144:153–167.

63. Caye A, Petresco S, de Barros AJD, Bressan RA, Gadelha A, Gonçalves H, et al. Relative Age and Attention-Deficit/Hyperactivity Disorder: Data From Three Epidemiological Cohorts and a Meta-analysis. J Am Acad Child Adolesc Psychiatry. 2020;59:990–997.

64. Nazar BP, Bernardes C, Peachey G, Sergeant J, Mattos P, Treasure J. The risk of eating disorders comorbid with attention-deficit/hyperactivity disorder: A systematic review and meta-analysis. Int J Eat Disord. 2016;49:1045–1057.

65. Cortese S, Moreira-Maia CR, St Fleur D, Morcillo-Peñalver C, Rohde LA, Faraone S V. Association between ADHD and obesity: A systematic review and meta-analysis. Am J Psychiatry. 2016;173:34–43.

66. Maher GM, O’Keeffe GW, Kearney PM, Kenny LC, Dinan TG, Mattsson M, et al. Association of Hypertensive Disorders of Pregnancy With Risk of Neurodevelopmental Disorders in Offspring: A Systematic Review and Meta-analysis. JAMA Psychiatry. 2018;75:809–819.

67. Zhu T, Gan J, Huang J, Li Y, Qu Y, Mu D. Association between perinatal hypoxic-ischemic conditions and attention-deficit/hyperactivity disorder: A meta-analysis. J Child Neurol. 2016;31:1235–1244.

68. Tseng PT, Yen CF, Chen YW, Stubbs B, Carvalho AF, Whiteley P, et al. Maternal breastfeeding and attention-deficit/hyperactivity disorder in children: a meta-analysis. Eur Child Adolesc Psychiatry. 2019;28:19–30.

69. Zeng Y, Tang Y, Tang J, Shi J, Zhang L, Zhu T, et al. Association between the different duration of breastfeeding and attention deficit/hyperactivity disorder in children: a systematic review and meta-analysis. Https://DoiOrg/101080/1028415X20181560905. 2018;23:811–823.

70. Birks L, Guxens M, Papadopoulou E, Alexander J, Ballester F, Estarlich M, et al. Maternal cell phone use during pregnancy and child behavioral problems in five birth cohorts. Environ Int. 2017;104:122–131.

71. Rowland J, Wilson CA. The association between gestational diabetes and ASD and ADHD: a systematic review and meta-analysis. Sci Reports 2021 111. 2021;11:1–16.

72. Zeng Y, Tang Y, Yue Y, Li W, Qiu X, Hu P, et al. Cumulative evidence for association of parental diabetes mellitus and attention-deficit/hyperactivity disorder. Neurosci Biobehav Rev. 2020;117:129–139.

73. Guo D, Ju R, Zhou Q, Mao J, Tao H, Jing H, et al. Association of maternal diabetes with attention deficit/hyperactivity disorder (ADHD) in offspring: A meta-analysis and review. Diabetes Res Clin Pract. 2020;165.

74. Zhao L, Li X, Liu G, Han B, Wang J, Jiang X. The association of maternal diabetes with attention deficit and hyperactivity disorder in offspring: a meta-analysis. Neuropsychiatr Dis Treat. 2019;15:675–684.

75. Yamamoto JM, Benham JL, Dewey D, Sanchez JJ, Murphy HR, Feig DS, et al. Neurocognitive and behavioural outcomes in offspring exposed to maternal pre-existing diabetes: a systematic review and meta-analysis. Diabetologia. 2019;62:1561–1574.

76. Polanska K, Kaluzny P, Aubert AM, Bernard JY, Duijts L, El Marroun H, et al. Dietary Quality and Dietary Inflammatory Potential During Pregnancy and Offspring Emotional and Behavioral Symptoms in Childhood: An Individual Participant Data Meta-analysis of Four European Cohorts. Biol Psychiatry. 2021;89:550–559.

77. Chen H, Qin L, Gao R, Jin X, Cheng K, Zhang S, et al. Neurodevelopmental effects of maternal folic acid supplementation: a systematic review and meta-analysis. Https://DoiOrg/101080/1040839820211993781. 2021. 2021. https://doi.org/10.1080/10408398.2021.1993781.

78. Zhu C, Jiang H, Sun J. Maternal infection during pregnancy and the risk of attention-deficit/hyperactivity disorder in the offspring: A systematic review and meta-analysis. Asian J Psychiatr. 2022;68:102972.

79. Li M, Francis E, Hinkle SN, Ajjarapu AS, Zhang C. Preconception and Prenatal Nutrition and Neurodevelopmental Disorders: A Systematic Review and Meta-Analysis. Nutrients. 2019;11.

80. Li L, Lagerberg T, Chang Z, Cortese S, Rosenqvist MA, Almqvist C, et al. Maternal pre-pregnancy overweight/obesity and the risk of attention-deficit/hyperactivity disorder in offspring: a systematic review, meta-analysis and quasi-experimental family-based study. Int J Epidemiol. 2020;49:857–875.

81. Jenabi E, Bashirian S, Khazaei S, Basiri Z. The maternal prepregnancy body mass index and the risk of attention deficit hyperactivity disorder among children and adolescents: a systematic review and meta-analysis. Korean J Pediatr. 2019;62:374–379.

82. Sanchez CE, Barry C, Sabhlok A, Russell K, Majors A, Kollins SH, et al. Maternal pre-pregnancy obesity and child neurodevelopmental outcomes: a meta-analysis. Obes Rev. 2018;19:464–484.

83. Manzari N, Matvienko-Sikar K, Baldoni F, O’Keeffe GW, Khashan AS. Prenatal maternal stress and risk of neurodevelopmental disorders in the offspring: a systematic review and meta-analysis. Soc Psychiatry Psychiatr Epidemiol. 2019;54:1299–1309.

84. Ge GM, Leung MTY, Man KKC, Leung WC, Ip P, Li GHY, et al. Maternal Thyroid Dysfunction During Pregnancy and the Risk of Adverse Outcomes in the Offspring: A Systematic Review and Meta-Analysis. J Clin Endocrinol Metab. 2020;105:3821–3841.

85. Levie D, Korevaar TIM, Mulder TA, Bath SC, Dineva M, Lopez-Espinosa MJ, et al. Maternal Thyroid Function in Early Pregnancy and Child Attention-Deficit Hyperactivity Disorder: An Individual-Participant Meta-Analysis. Https://HomeLiebertpubCom/Thy. 2019;29:1316–1326.

86. Thompson W, Russell G, Baragwanath G, Matthews J, Vaidya B, Thompson-Coon J. Maternal thyroid hormone insufficiency during pregnancy and risk of neurodevelopmental disorders in offspring: A systematic review and meta-analysis. Clin Endocrinol (Oxf). 2018;88:575–584.

87. Jenabi E, Seyedi M, Bashirian S, Fereidooni B. Is there an association between labor induction and attention-deficit/hyperactivity disorder among children? Clin Exp Pediatr. 2021;64:489–493.

88. Lonfeldt NN, Verhulst FC, Strandberg-Larsen K, Plessen KJ, Lebowitz ER. Assessing risk of neurodevelopmental disorders after birth with oxytocin: a systematic review and meta-analysis. Psychol Med. 2019;49:881–890.

89. Robinson R, Girchenko P, Pulakka A, Heinonen K, Lähdepuro A, Lahti-Pulkkinen M, et al. ADHD symptoms and diagnosis in adult preterms: systematic review, IPD meta-analysis, and register-linkage study. Pediatr Res 2022. 2022:1–11.

90. Anderson PJ, de Miranda DM, Albuquerque MR, Indredavik MS, Evensen KAI, Van Lieshout R, et al. Psychiatric disorders in individuals born very preterm / very low-birth weight: An individual participant data (IPD) meta-analysis. EClinicalMedicine. 2021;42.

91. Franz AP, Bolat GU, Bolat H, Matijasevich A, Santos IS, Silveira RC, et al. Attention-Deficit/Hyperactivity Disorder and Very Preterm/Very Low Birth Weight: A Meta-analysis. Pediatrics. 2018;141.

92. Momany AM, Kamradt JM, Nikolas MA. A Meta-Analysis of the Association Between Birth Weight and Attention Deficit Hyperactivity Disorder. J Abnorm Child Psychol. 2018;46:1409–1426.

93. Allotey J, Zamora J, Cheong-See F, Kalidindi M, Arroyo-Manzano D, Asztalos E, et al. Cognitive, motor, behavioural and academic performances of children born preterm: a meta-analysis and systematic review involving 64 061 children. BJOG An Int J Obstet Gynaecol. 2018;125:16–25.

94. Hemmi MH, Wolke D, Schneider S. Associations between problems with crying, sleeping and/or feeding in infancy and long-term behavioural outcomes in childhood: a meta-analysis. Arch Dis Child. 2011;96:622–629.

95. Li SL, Kam KW, Chee ASH, Zhang XJ, Chen LJ, Yip WWK, et al. The association between attention-deficit/hyperactivity disorder and retinal nerve fiber/ganglion cell layer thickness measured by optical coherence tomography: a systematic review and meta-analysis. Int Ophthalmol. 2021;41:3211–3221.

96. Russell AE, Ford T, Williams R, Russell G. The Association Between Socioeconomic Disadvantage and Attention Deficit/Hyperactivity Disorder (ADHD): A Systematic Review. Child Psychiatry Hum Dev. 2016;47:440–458.

97. Nayeri T, Sarvi S, Moosazadeh M, Hosseininejad Z, Amouei A, Daryani A. Toxoplasma gondii infection and risk of attention-deficit hyperactivity disorder: a systematic review and meta-analysis. Https://DoiOrg/101080/2047772420201738153. 2020;114:117–126.

98. Asarnow RF, Newman N, Weiss RE, Su E. Association of Attention-Deficit/Hyperactivity Disorder Diagnoses With Pediatric Traumatic Brain Injury: A Meta-analysis. JAMA Pediatr. 2021;175:1009–1016.

99. Adeyemo BO, Biederman J, Zafonte R, Kagan E, Spencer TJ, Uchida M, et al. Mild Traumatic Brain Injury and ADHD: A Systematic Review of the Literature and Meta-Analysis. J Atten Disord. 2014;18:576–584.
